# BRCA2 suppresses replication stress-induced mitotic and G1 abnormalities through homologous recombination

**DOI:** 10.1038/s41467-017-00634-0

**Published:** 2017-09-13

**Authors:** Weiran Feng, Maria Jasin

**Affiliations:** 1 Developmental Biology Program, Memorial Sloan Kettering Cancer Center, 1275 York Avenue, New York, NY 10065 USA; 2 Louis V. Gerstner Jr. Graduate School of Biomedical Sciences, Memorial Sloan Kettering Cancer Center, 1275 York Avenue, New York, NY 10065 USA

## Abstract

Mutations in the tumor suppressor *BRCA2* predominantly predispose to breast cancer. Paradoxically, while loss of BRCA2 promotes tumor formation, it also causes cell lethality, although how lethality is triggered is unclear. Here, we generate *BRCA2* conditional non-transformed human mammary epithelial cell lines using CRISPR-Cas9. Cells are inviable upon BRCA2 loss, which leads to replication stress associated with under replication, causing mitotic abnormalities, 53BP1 nuclear body formation in the ensuing G1 phase, and G1 arrest. Unexpected from other systems, the role of BRCA2 in homologous recombination, but not in stalled replication fork protection, is primarily associated with supporting human mammary epithelial cell viability, and, moreover, preventing replication stress, a hallmark of pre-cancerous lesions. Thus, we uncover a DNA under replication-53BP1 nuclear body formation-G1 arrest axis as an unanticipated outcome of homologous recombination deficiency, which triggers cell lethality and, we propose, serves as a barrier that must be overcome for tumor formation.

## Abstract (web summary)

BRCA2 mutations promote tumour formation while also paradoxically causing cell lethality. Here the authors generate conditional BRCA2 loss in a non-transformed human mammary cell line and see increased replication stress due to under-replication of DNA.

## Introduction

Monoallelic inheritance of a deleterious mutation in the *BRCA1* or *BRCA2* tumor suppressor confers susceptibility to breast and ovarian cancer^1^. Biallelic mutations of *BRCA2* are also linked to Fanconi anemia, a syndrome characterized by developmental issues and tumor predisposition^2^. BRCA2 suppresses genome instability, a hallmark of cancer, by playing a central role in two processes: homologous recombination (HR) for the repair of DNA lesions and protection of nascent strands at stalled replication forks from degradation^3^.

HR is the best-characterized function of BRCA2, where it loads the RAD51 recombinase onto single-stranded DNA (ssDNA), which form a nucleoprotein filament to mediate homologous strand exchange^3^. This process is responsible for repairing DNA double-strand breaks (DSBs), which may include those generated by replication fork breakdown^4^. Due to impaired HR, BRCA2-deficient cells are hypersensitive to agents that cause DSBs, such as cross-linking agents and poly (ADP-ribose) polymerase (PARP) inhibitors. These sensitivities are being exploited in therapeutic approaches. Replication fork protection prevents degradation of nascent DNA strands at stalled replication forks by the MRE11 nuclease and requires BRCA1 and other Fanconi anemia proteins, as well as BRCA2^5–7^. Recently, MRE11 recruitment to stalled replication forks has been shown to be mediated by a number of proteins, including PARP1^8, 9^. HR and replication fork protection are functionally separable processes, despite sharing a requirement of key proteins^5, 6, 8, 9^.

Loss of the wild-type *BRCA2* allele, indicative of functional inactivation of BRCA2, is common in breast and ovarian cancers arising in *BRCA2* mutation carriers. Conditional knockout of BRCA2 in mouse models also results in tumorigenesis^10, 11^. However, rather than providing a growth advantage as in cancers, BRCA2 deficiency causes inviability of mouse embryos and normal mouse cells^12–15^, although it is not fully understood how lethality is induced in the absence of BRCA2 in otherwise normal cells and how tumor cells emerge and survive the crisis when BRCA2 is lost, which may potentially impact therapeutic approaches.

Recently, the role of BRCA2 in the protection of stalled replication forks was reported to be sufficient to sustain viability of mouse embryonic stem (ES) cells and to confer resistance of tumor cells to crosslinking agents and PARP inhibitors even in the absence of functional HR^8, 9^. However, although viable, these ES cells grow poorly, and fork protection alone is not capable of supporting embryo development^8^, suggesting that HR is essential in some contexts. How the two pathways functionally interact to ensure genome integrity and cell viability in adult tissues, such as normal mammary cells to prevent breast cancer initiation remains elusive.

To dissect the mechanisms by which relatively normal, non-cancerous mammary cells respond to BRCA2 deficiency, we developed conditional cell lines to examine the acute response to BRCA2 loss. We demonstrate that BRCA2 deficiency triggers replication stress that is transmitted to the next cell cycle through DNA under replication, which causes chromosome missegregation, forming 53BP1 nuclear bodies at G1. p53-dependent G1 arrest and senescence are activated, ultimately leading to cell inviability. Moreover, using multiple separation-of-function approaches, we show that HR, but not protection of stalled replication forks, is primarily responsible for suppressing replication stress and supporting cell viability. Thus, our work reveals G1 abnormalities as an unanticipated mechanism to trigger cell lethality upon BRCA2 deficiency. We propose HR as the major pathway to guard against replication stress, a hallmark of precancerous lesions.

## Results

### BRCA2 is essential for human mammary MCF10A cell viability

**Fig. 1:**
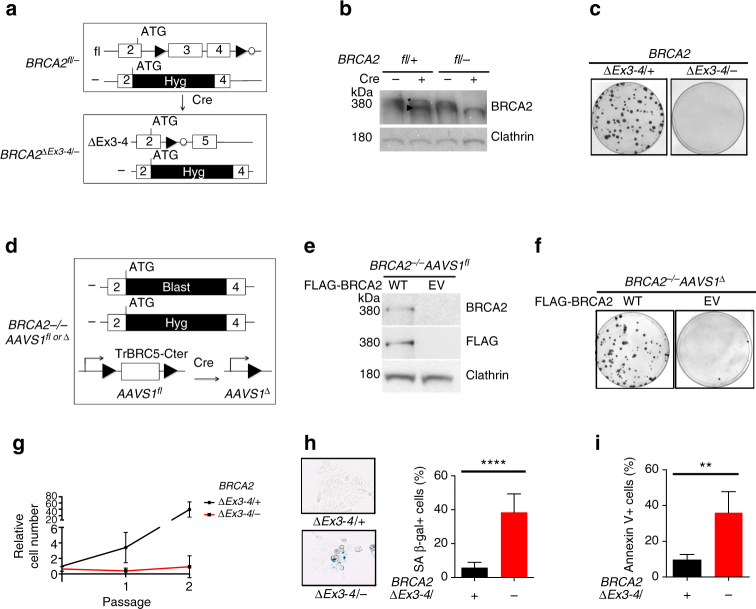
BRCA2 is essential for non-transformed human mammary MCF10A cell viability. **a** Schematic of the *BRCA2* exon3-4-floxed conditional system in MCF10A cells (*filled triangle*, loxP site; *open circle*, FRT site; Hyg, hygromycin-resistance gene). **b** Western blot of *BRCA2* exon3-4-floxed cell extracts with or without Cre expression (*asterisk*, full-length BRCA2; *arrowhead*, ∆Ex3-4 peptide). The BRCA2 antibody Ab-1 detects BRCA2 amino acids 1651–1821. **c** *BRCA2* ^*∆Ex3-4/−*^ cells were plated for clonogenic survival. Representative plates are shown. **d** Schematic of the *BRCA2* ^*−/−*^ *AAVS1* ^*fl*^ conditional system in MCF10A cells. Blast, Blasticidin-resistance gene. **e** Western blot showing BRCA2 expression in stably complemented *BRCA2* ^*−/−*^ *AAVS1* ^*fl*^ cells (*WT*, wild-type BRCA2; *EV*, empty vector). **f** *BRCA2* ^*−/−*^ *AAVS1* ^*∆*^ cells were plated for clonogenic survival. **g** *BRCA2* ^*∆Ex3-4/−*^ cells were serially passaged every 3 days. Cell number was determined at the end of each passage and normalized to the number of *BRCA2* ^*∆Ex3-4/+*^ cells at passage 0. **h** Cells were stained for senescence-associated β-galactosidase (SA β-gal). *Left*: representative images; *Right*: comparison of the percent SA β-gal+ cells. **i** Cells were quantified for apoptosis using Annexin V staining. *Error bars* in this figure represent one standard deviation from the mean (s.d.). *n* > 3. **p < 0.01; ****p < 0.0001 (unpaired two-tailed *t*-test)

To better understand BRCA2’s role in a tumor-relevant cell type, we generated a *BRCA2* conditional system in MCF10A cells, a non-transformed human mammary epithelial cell line with a relatively stable genome^16^. Through CRISPR-Cas9-mediated gene targeting, we knocked in loxP sites to flank exons 3 and 4 of one *BRCA2* allele, and knocked out the other allele by targeting a selectable marker immediately downstream of the start codon (Fig. 1a, Supplementary Fig. 1a–d). Deletion of exons 3 and 4 is expected to cause a frameshift mutation that generates a pre-mature stop codon to prevent further protein translation. Moreover, exons 3 and 4 encode residues that are essential for PALB2 binding^17^, which is required for mouse embryonic stem cell viability^18^. An exon 3 skipping mutation is associated with familial breast cancer^19^, further supporting the notion that loss of PALB2 binding disrupts BRCA2 function.

BRCA2 inactivation in these conditional cells was achieved by infecting *BRCA2* ^*fl/−*^ cells with either adeno-Cre or a lentivirus that expresses a self-deleting Cre^20^. We detected the expression of a peptide smaller than full-length BRCA2 in the resulting *BRCA2* ^*∆Ex3-4/−*^ cells as well as in control *BRCA2* ^*∆Ex3-4/+*^ cells (Fig. 1b). Transcript analysis indicated aberrant splicing that presumably promotes translation from a downstream, in-frame start codon (Supplementary Fig. 1e). PALB2 binding mediates BRCA2 chromatin localization; indeed, the truncated ∆Ex3-4 peptide was found to be deficient in chromatin binding (Supplementary Fig. 1f, g). To test whether exon 3-4 deletion affected viability of MCF10A cells, we performed clonogenic survival assays after Cre expression. Unlike *BRCA2* ^*∆Ex3-4/+*^ cells, *BRCA2* ^*∆Ex3-4/−*^ cells did not form colonies (Fig. 1c), indicating that intact BRCA2 is essential for the viability of these non-transformed human mammary epithelial cells.

We also generated a second *BRCA2* conditional system in which BRCA2 is completely lost upon Cre expression by targeting a floxed *BRCA2* transgene (*TrBRC5-Cter*)^21^ transgene into the safe-harbor *AAVS1* locus. The endogenous *BRCA2* alleles were then knocked out by targeting selectable markers downstream of the start codon to generate *BRCA2* ^*−/−*^ *AAVS1* ^*fl*^ cells (Fig. 1d, Supplementary Fig. 2). The TrBRC5-Cter peptide restores some BRCA2 function^21^, although it is expressed at low levels in the *BRCA2* ^*−/−*^ *AAVS1* ^*fl*^ cells (Supplementary Fig. 2c) and so the cells grow slowly. The requirement for BRCA2 was studied by introducing a vector that expresses full-length, FLAG-tagged BRCA2 (WT) or an empty vector (EV). Unlike the WT-complemented cells, the EV-transfected *BRCA2* ^*−/−*^ *AAVS1* ^*fl*^ cells were devoid of full-length BRCA2 (Fig. 1e). Only the WT-complemented cells formed viable clones upon Cre expression (Fig. 1f). Thus, the AAVS1 system recapitulated our observations from the ∆Ex3-4 system.

Consistent with cell inviability, BRCA2 deficiency led to an acute proliferation defect within the first few passages after Cre infection (Fig. 1g) associated with cellular senescence and apoptosis (Fig. 1h, i). Because no viable BRCA2-deficient clones were obtained from either system, unless otherwise noted, we performed our analysis of BRCA2-deficient cells shortly after Cre expression.

### Fork protection is a minor survival and repair pathway

BRCA2 protects genome integrity through a well-established role in HR and a more recently described HR-independent role in the protection of stalled replication forks^5^. To examine HR levels in the BRCA2-deficient human mammary cells, we used the stably integrated DR-GFP reporter that produces functional GFP only when a DSB introduced into the reporter is repaired through HR^22^. As expected, the BRCA2-deficient cells showed a dramatic reduction in HR repair of the DSB (**~**10-fold, Supplementary Fig. 3a). One hallmark of HR deficiency is hypersensitivity to cross-linking agents (e.g., cisplatin) and PARP inhibitors (e.g., olaparib) and mild sensitivity to irradiation (IR). In line with this view, treatment with either cisplatin or olaparib led to substantially higher levels of unrepaired DNA damage in *BRCA2* ^*∆Ex3-4/−*^ cells compared with *BRCA2* ^*∆Ex3-4/+*^ cells, as measured by the nuclear intensity of γH2AX (Supplementary Fig. 3b, c). In addition, while both *BRCA2* genotypes displayed similar initial levels of IR-induced γH2AX, *BRCA2* ^*∆Ex3-4/−*^ cells showed slower repair kinetics (Supplementary Fig. 3d). The delayed repair was more pronounced in the S/G2 phases compared with G1, which is consistent with the cell-cycle phase preference for HR repair. Altogether, these results confirm that these human mammary cells have a severe HR deficiency upon BRCA2 deficiency.

**Fig. 2:**
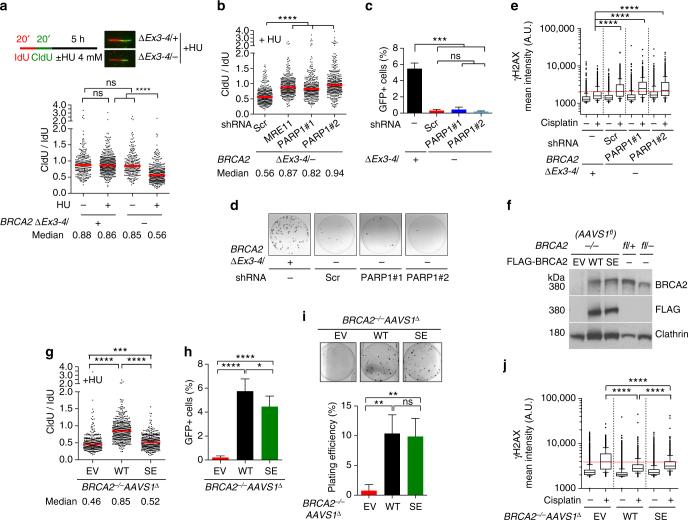
Fork protection plays a minor role in cell viability and DNA repair. **a** DNA fiber analysis to quantify fork protection. Schematic of the experimental design and representative images are shown in the *inset*. Median CldU/IdU tract length ratios are indicated in the graph (*red bars*). *Graphs* here and below represent the pooled results of > 200 fibers per genotype from at least two independent experiments, analyzed by a two-tailed Mann–Whitney test. **b** Cells stably expressing the indicated shRNAs were analyzed for fork protection in the presence of HU by a two-tailed Mann–Whitney test. Scr, scrambled shRNA. **c** HR analysis. PARP1 knockdown cells were infected with I-SceI-expressing lentivirus and the percent GFP+ cells was analyzed by an unpaired two-tailed *t*-test. *n* = 3. **d** PARP1 knockdown cells were plated for clonogenic survival. Residual colonies from *BRCA2* ^*∆Ex3-4/−*^ plates were all were confirmed by PCR to have maintained the *BRCA2* ^*fl/−*^ genotype (i.e., escaped Cre recombination). **e** Cisplatin-induced γH2AX. PARP1 knockdown cells were treated with 5 µM cisplatin for 5 h and released for another 24 h before analysis. γH2AX mean nuclear intensities of >1000 individual cells are shown from one experiment, which is representative of three independent experiments, analyzed by a two-tailed Mann–Whitney test. The *dotted red line* indicates the median of *BRCA2* mutant cells treated with the scrambled shRNA exposed to cisplatin. A.U., arbitrary units. *Box* and *whiskers* show the 10th and 90th percentiles. **f** Western blotting of *BRCA2* ^*−/−*^ *AAVS1* ^*fl*^ cells stably expressing BRCA2 WT or BRCA2 SE. **g** DNA fiber analysis to quantify fork protection in the presence of HU. Median CldU/IdU tract length ratios are indicated (*red bars*), analyzed by a two-tailed Mann–Whitney test. **h** HR analysis, as in **c**. *n* > 3. **i** Clonogenic survival analysis using an unpaired two-tailed *t*-test. *n* = 3. **j** Cisplatin-induced γH2AX analysis, as in **e**. The *dotted red line* indicates the median of the *BRCA2* ^*−/−*^ *AAVS1* ^*∆*^ cells exposed to cisplatin. *Error bars* s.d. ns, not significant; *p < 0.05; **p < 0.01; ***p < 0.001; ****p < 0.0001

We next performed DNA fiber assays to confirm that the BRCA2-deficient cells show degradation of nascent DNA strands at stalled replication forks that is dependent on MRE11 nuclease^5^. We sequentially labeled the cells with a pulse of IdU (red), followed by CldU (green), which is preferentially lost in the absence of fork protection upon replication stress, in this case from the fork stalling agent hydroxyurea (HU) (Fig. 2a). HU treatment triggered a substantially lower relative CldU tract length in BRCA2-deficient cells compared to control cells expressing wild-type BRCA2, indicating nascent strand degradation (Fig. 2a). As expected, replication fork degradation was dependent upon MRE11 nuclease (Fig. 2b, Supplementary Fig. 4a). As a complementary approach, cells were treated with a single IdU pulse (red) before HU treatment. Again, BRCA2-deficient cells showed considerably shortened nascent strands (IdU-labeled) after HU treatment (Supplementary Fig. 4b), confirming that BRCA2 protects stalled forks. In addition to depletion of MRE11 itself, PARP1 deficiency was recently shown to rescue fork protection, as PARP1 mediates MRE11 chromatin recruitment during replication stress, but not HR^8, 9^, which we also observed (Fig. 2b, c, Supplementary Fig. 4c, d).

Restoration of nascent DNA strand stability at stalled forks was shown recently to be sufficient to confer viability to *Brca2* null mouse ES cells and cisplatin resistance to BRCA2-deficient mouse B cells and a human tumor cell line^8, 9, 23^. Surprisingly, PARP1 depletion failed to restore viability to the BRCA2-deficient MCF10A cells (Fig. 2d). Moreover, PARP1 depletion failed to suppress cisplatin-induced γH2AX formation (Fig. 2e).

As these results are contrary to those seen in other systems, we also generated *PARP1* knockouts in MCF10A cells using CRISPR-Cas9. Although both PARP1 heterozygosity and complete knockout restored fork protection, but not HR, to the BRCA2-deficient cells, neither restored cell viability (Supplementary Fig. 5a–d). We also examined the effects of PARP inhibition and MRE11 depletion, which were also previously shown^5, 8^, and confirmed in our system, to prevent nascent strand degradation (Fig. 2b, Supplementary Fig. 5e). Again, neither treatment conferred a growth advantage to BRCA2-deficient cells (Supplementary Fig. 5f, g). Thus, in contrast to previous observations in mouse and tumor cells, our results suggest that fork protection is not sufficient to support cell viability or repair crosslink-induced DNA damage in these non-transformed human mammary epithelial cells.

We next asked whether protection of nascent DNA strand at stalled forks is necessary for cell survival and DNA repair. In hamster cells, mutating BRCA2 S3291 (S3291A) specifically abrogates replication fork protection without affecting HR^5^, thus providing a separation of function mutation to distinguish the two functions. In this study, we investigated the S3291E mutation, which, like S3291A, also disrupts RAD51 binding to the BRCA2 C terminus^24^. To this end, we expressed the BRCA2 S3291E (BRCA2 SE) mutant at physiological levels in both BRCA2 conditional systems (*BRCA2* ^*−/−*^ *AAVS1* ^*∆*^, Fig. 2f; *BRCA2* ^*∆Ex3-4/−*^, Supplementary Fig. 6a).

As expected, BRCA2 SE-complemented cells in both systems showed nascent strand degradation during HU treatment but only a mild or moderate HR defect (Fig. 2g, h, Supplementary Fig. 6b–d). Notably, BRCA2 SE-expressing cells were capable of forming colonies (Fig. 2i, Supplementary Fig. 6e, f), demonstrating the ability of cells to proliferate in the absence of fork protection. In the *BRCA2* ^*−/−*^ *AAVS1* ^*∆*^ system, colony number was fully restored by BRCA2 SE expression. In *BRCA2* ^*∆Ex3-4/−*^ cells, colony number was also significantly restored with BRCA2 SE expression and colonies were as large as those with BRCA2 WT. Nonetheless, plating efficiency of these cells was still reduced relative to WT-complemented cells, which may be related to the lack of full restoration of HR (see below), possibly due to slightly lower expression of BRCA2 SE, when compared either to BRCA2 WT in *BRCA2* ^*∆Ex3-4/−*^ cells or to BRCA2 SE in *BRCA2* ^*−/−*^ *AAVS1* ^*∆*^ cells (Supplementary Fig. 6a, g), although interference from the BRCA2^∆Ex3-4^ peptide cannot be ruled out. Furthermore, BRCA2 SE substantially suppressed cisplatin-induced DNA damage formation in both systems, with only a marginal defect compared to BRCA2 WT (Fig. 2j, Supplementary Fig. 6h). Thus, protection of stalled replication forks is dispensable for cell survival and only plays a minor role in repairing cisplatin-induced DNA damage.

### BRCA2 ablation causes spontaneous DNA damage and G1 arrest

**Fig. 3:**
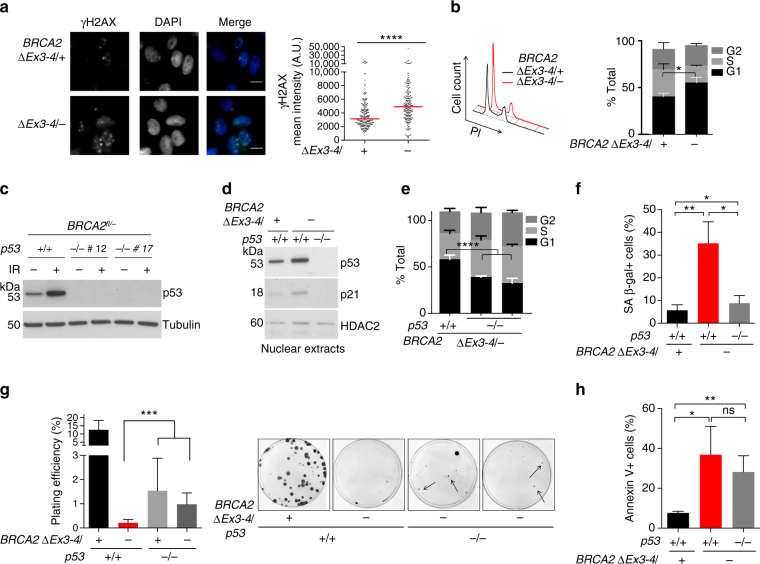
BRCA2 deficiency triggers spontaneous DNA damage and G1 arrest. **a** *BRCA2* ^*∆Ex3-4/−*^ cells were immunostained for γH2AX. DNA was counterstained with DAPI. Representative images (*left*) and quantification of γH2AX mean nuclear intensity (*right*) are shown with analysis by a two-tailed Mann–Whitney test. Median γH2AX intensity, *red bars*. *Scale bars* 10 µm. **b** Cell cycle analysis demonstrates an increase in the G1 fraction upon BRCA2 deficiency. Representative plots displaying results from the same amount of cells for both samples are shown on the *left*. An unpaired two-tailed *t*-test was used for the analysis. *n* ≥ 3. **c** Western blot showing p53 loss. Cells were harvested 2 h after 10 Gy irradiation (IR). Two independent *BRCA2* ^*fl/−*^ *p53* ^*−/−*^ clones were analyzed. **d** Western blots of nuclear extracts prepared from the indicated cells. **e** Cell cycle analysis. An unpaired two-tailed *t*-test was used for the analysis. *n* ≥ 3. **f** Cellular senescence, as indicated by SA β-gal staining. An unpaired two-tailed *t*-test was used for the analysis. *n* ≥ 3. **g** p53 loss leads to a partial increase in clonogenic survival of BRCA2-deficient cells. *Left*: plating efficiency, as analyzed by an unpaired two-tailed *t*-test. *n* ≥ 3. *Right*: images of representative plates. *Arrows* highlight typical PCR-validated *BRCA2* ^*∆Ex3-4/−*^ *p53* ^*−/−*^ colonies. **h** Apoptosis analysis using Annexin V staining. Analysis is by an unpaired two-tailed *t*-test. *n* ≥ 3. *Error bars* s.d. ns, not significant; *p < 0.05; **p < 0.01; ***p < 0.001; ****p < 0.0001

To gain more insight into how cell lethality is triggered in these non-transformed, human mammary epithelial cells, we analyzed the consequences of BRCA2 deficiency at the cellular level. As expected, γH2AX staining under unchallenged conditions revealed a higher level of spontaneous DNA damage in *BRCA2* ^*∆Ex3-4/−*^ cells (Fig. 3a, Supplementary Fig. 3b, c). DNA damage activates checkpoints to pause cell cycle progression until DNA repair is complete^25^. Given its roles in replication fork protection and DNA repair during the S and G2 phases, BRCA2-deficient cells would be expected to be arrested in these cell cycle phases^13^. Surprisingly, however, cell cycle analysis demonstrated that *BRCA2* ^*∆Ex3-4/−*^ cells were enriched in G1 instead (Fig. 3b, Supplementary Fig. 7).

To test whether p53 is responsible for G1 arrest and inviability of BRCA2-deficient cells, we generated *p53* knock out cells in the *BRCA2* ^*fl/−*^ background using CRISPR-Cas9 (Fig. 3c). *BRCA2* ^*∆Ex3-4/−*^ cells exhibited an increase in p53 levels and p53-dependent p21 induction compared to *BRCA2* ^*∆Ex3-4/+*^ cells (Fig. 3d), indicating p53 pathway activation. Importantly, the *BRCA2* ^*∆Ex3-4/−*^ G1 cell population was diminished upon p53 loss (Fig. 3e, Supplementary Fig. 7). p53 loss also abrogated cellular senescence induced by BRCA2 deficiency (Fig. 3f), in agreement with a previous study using mouse cells^26^. Remarkably, PCR-validated *BRCA2* ^*∆Ex3-4/−*^ colonies were formed only in the absence of p53 (Fig. 3g, Supplementary Fig. 8). These colonies were smaller and fewer compared to the *BRCA2* ^*∆Ex3-4/+*^ control and grew very slowly upon expansion, indicating only a partial rescue of cell viability by p53 loss. Interestingly, the high apoptotic fraction of *BRCA2* ^*∆Ex3-4/−*^ cells was not affected by p53 loss (Fig. 3h), thus explaining the partial rescue. Collectively, our results suggest that p53 pathway activation, likely in response to spontaneous DNA damage, leads to G1 arrest and cellular senescence and contributes to cell lethality upon BRCA2 deficiency.

### BRCA2 suppresses G1 53BP1 nuclear body formation

**Fig. 4:**
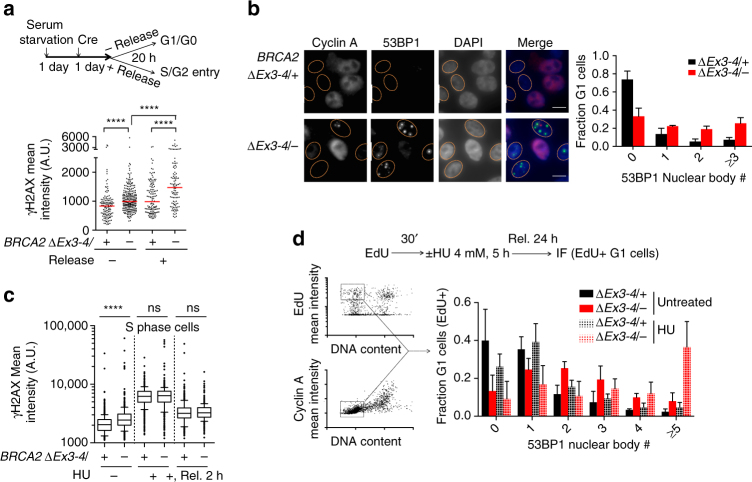
BRCA2 suppresses replication stress associated with G1 53BP1 nuclear bodies. **a** *BRCA2* ^*∆Ex3-4/–*^ cells were serum starved with or without release, as in the schematic (*top*), and γH2AX mean nuclear intensities were quantified (*bottom*). γH2AX mean nuclear intensities are shown from one experiment, which is representative of two independent experiments. Median γH2AX intensity, *red bars*. **b** 53BP1 nuclear body analysis in G1 phase. Cyclin A–nuclei (indicating G1 phase) are outlined (*left*). Quantification of 53BP1 nuclear body distribution is shown (*right*). *n* = 4. *Scale bars* 10 µm. **c** S phase DNA damage analysis. Where indicated, cells were treated with EdU for 30 min followed by HU treatment (4 mM, 5 h) with or without release (Rel.) before harvest. Quantification of γH2AX mean nuclear intensities is shown from one experiment, which is representative of two independent experiments. *Box* and *whiskers* show the 10th and 90th percentiles. **d** HU-induced 53BP1 nuclear body formation analysis, as in the schematic (*top*). Sample plots for high content image cytometry analysis are shown on the *left*. The EdU+ G1 cell fraction (i.e., EdU+, cyclin A–, 1N DNA content) for 53BP1 nuclear body quantification is highlighted. Quantification of 53BP1 nuclear body distribution in EdU+ cells in G1 at the time of harvest is shown in the graph. *n* ≥ 3. *Error bars* s.d. ns, not significant; ****p < 0.0001 (two-tailed Mann–Whitney test)

To investigate why BRCA2 deficiency caused G1 cell cycle arrest, we first sought to determine the cell cycle stage at which spontaneous DNA damage arose. γH2AX induction was associated with S and G2 entry of the BRCA2-deficient cells after release from arrest and was also enriched within the S/G2 population specifically marked by cyclin A (Fig. 4a, Supplementary Fig. 9), indicating that spontaneous DNA damage primarily originates in these cell cycle phases.

53BP1 nuclear bodies mark DNA lesions in G1 as a consequence of replication stress in the previous cell cycle^27, 28^. We hypothesized that the G1 arrest arising from BRCA2 deficiency may be associated with 53BP1 nuclear body formation arising from lesions generated in the previous S/G2 phases. Indeed, spontaneously arising 53BP1 nuclear bodies were dramatically induced in number in G1 phase *BRCA2* ^*∆Ex3-4/−*^ cells (i.e., cyclin A–cells, Fig. 4b). The majority of G1 *BRCA2* ^*∆Ex3-4/−*^ cells had 53BP1 nuclear bodies, with ~20% showing three or more, whereas 53BP1 nuclear bodies were rare in control G1 cells.

We next determined the impact of replication stress on BRCA2-deficient cells. While HU treatment greatly increased the amount of damage, it did not have a specific impact on overall DNA damage induction or recovery in BRCA2-deficient cells during S phase (Fig. 4c). By contrast, however, HU treatment led to a remarkable induction of 53BP1 nuclear body formation in the next G1 phase, as revealed by high-content image cytometry (Fig. 4d). Thus, in BRCA2-deficient cells, HU-generated replication stress does not induce a more profound DNA damage response in S phase, but rather in the subsequent G1 phase. Because 53BP1 interacts with p53 and 53BP1 nuclear bodies mark DNA lesions and contain classical DNA damage signaling proteins^28–30^, 53BP1 nuclear bodies can conceivably trigger the aforementioned p53-dependent G1 arrest.

### BRCA2 prevents under replication and mitotic abnormalities

**Fig. 5:**
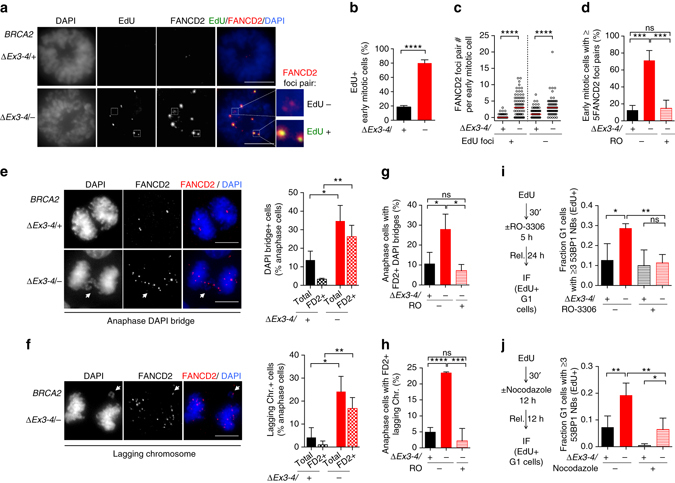
BRCA2 deficiency causes DNA under replication that results in abnormal mitoses. **a**–**c** *BRCA2* ^*∆Ex3-4/–*^ cells were released for 22 h from serum starvation to increase mitotic cells, incubated with EdU for 1 h, and then analyzed for mitotic DNA synthesis. Early mitotic cells defined as being in prophase, prometaphase, or metaphase were analyzed for EdU foci that co-localize with FANCD2 foci pairs. **a** Representative images of mitotic DNA synthesis. *Scale bars* 10 µm. **b** Percent early mitotic cells containing EdU foci, analyzed by an unpaired two-tailed *t*-test. *n* = 3. **c** FANCD2 foci pairs with or without EdU foci co-localization. *Graphs* represent the pooled results of three independent experiments, each analyzed by a two-tailed Mann–Whitney test. Median FANCD2 foci pair number, *red bars*. **d** FANCD2 foci pair analysis in early mitotic cells. Cells were untreated, or treated with the CDK1 inhibitor RO-3306 (10 µM, 24 h) to delay mitotic entry, and released for 1 h before analysis of FANCD2 foci pairs in early mitotic cells. Analysis is by an unpaired two-tailed *t*-test. *n* = 4. **e**–**h** Anaphase cells were analyzed for DAPI bridges (**e**, **g**) and lagging chromosomes (**f**, **h**). RO-3306 treatment (10 µM, 24 h with release for 1 h) was applied where indicated. Representative deconvolved images are shown on the *left* of **e** and **f**. *n* = 3. Statistical analysis was by an unpaired two-tailed *t*-test. FD2, FANCD2. *Scale bars* 10 µm. **i**, **j**. 53BP1 nuclear body analysis with RO-3306 (10 µM, **i**) or nocodazole (100 ng ml^−1^, **j**) treatment and released as in the schematic on the *left*. The fraction of EdU+ cells in G1 at the time of harvest that contains ≥3 53BP1 nuclear bodies (NBs) is shown in the graph on the *right*. Analysis is by an unpaired two-tailed *t*-test. *n* ≥ 3. *Error bars* s.d. ns, not significant; *p < 0.05; **p < 0.01; ***p < 0.001; ****p < 0.0001

One possible reason for 53BP1 nuclear body formation is that it serves as a response to replication stress that interferes with the timely completion of replication: The resulting under-replicated DNA forms unresolved structures, which cause aberrations during mitosis and ultimately generate 53BP1 nuclear bodies in the daughter cells^31^. To delineate the impact of BRCA2 inactivation on these processes, we first analyzed DNA under replication using mitotic DNA synthesis as a surrogate, a pathway activated during early stages of mitosis as a compensatory attempt to finish replication of unduplicated DNA^32^. Foci of DNA synthesis were evident in a majority of M-phase BRCA2-deficient cells (> 70%) and were substantially elevated in number, while they were rarely present in control cells (Fig. 5a, b, Supplementary Fig. 10a). Notably, mitotic DNA synthesis occurred almost exclusively at sites marked by FANCD2 foci pairs (Fig. 5a), an indicator of incompletely replicated DNA in the preceding S phase^33, 34^. Overall, the total number of FANCD2 foci pairs was greatly elevated with BRCA2 deficiency, and a substantial fraction of these were sites of DNA synthesis (Fig. 5c, d). These results imply that BRCA2 suppresses DNA under replication. Moreover, that BRCA2-deficient cells also exhibited a concomitant elevation in the number of FANCD2 foci pairs in which DNA synthesis did not occur (Fig. 5a, c) suggests that incompletely replicated DNA may not be fully duplicated during early mitosis and is, therefore, likely carried over to later mitotic stages.

Unresolved DNA structures such as persistent under-replicated DNA can cause chromosome non-disjunction manifested as ultra-fine bridges (UFBs), a DNA linkage that stains negative for conventional DNA dyes (e.g., DAPI) but can be visualized by staining with bound proteins such as PICH^35, 36^. These DNA linkages can in turn cause chromosome missegregation, forming DAPI+ anaphase bridges and lagging chromosomes, ultimately generating micronuclei^33, 34, 37, 38^. Consistent with this, UFBs, anaphase bridges, and lagging chromosomes were all significantly more common in the BRCA2-deficient cells (Fig. 5e, f, Supplementary Fig. 10b). Importantly, these anaphase structures were associated with FANCD2 foci in most cases (Fig. 5e, f, Supplementary Fig. 10b), suggesting that they form as a consequence of DNA under replication. To test this hypothesis, cells were allowed more time to finish replication before entering mitosis by treatment with the CDK1 inhibitor RO-3306, which arrests cells at G2/M phase^39^. As expected, FANCD2 foci pairs in early mitotic BRCA2-deficient cells were diminished to control levels upon release from RO-3306 treatment (Fig. 5d). Moreover, formation of both anaphase DAPI bridges and lagging chromosomes in these cells were also abrogated (Fig. 5g, h). BRCA2 deficiency also led to a concomitant increase of micronuclei (Supplementary Fig. 10c). These results strongly suggest that DNA under replication leads to mitotic abnormalities upon BRCA2 deficiency.

Next, we examined if DNA under replication is also the cause of the observed 53BP1 nuclear bodies, using a pulse of EdU to mark S-phase cells before RO-3306 treatment (Fig. 5i). 53BP1 nuclear body formation in EdU+ *BRCA2* ^*∆Ex3-4/−*^ cells was diminished to control levels upon release into the next G1 phase. Similarly, nocodazole, which leads to a prolonged prometaphase during which the compensatory mitotic DNA synthesis occurs^32^, also abolished the subsequent 53BP1 nuclear body formation (Fig. 5j). Taken together, our data strongly suggest that BRCA2 suppresses DNA under replication, which, if unrestrained, causes mitotic abnormalities that lead to 53BP1 nuclear body formation in the subsequent G1 phase.

### Replication stress suppression primarily associates with HR

**Fig. 6:**
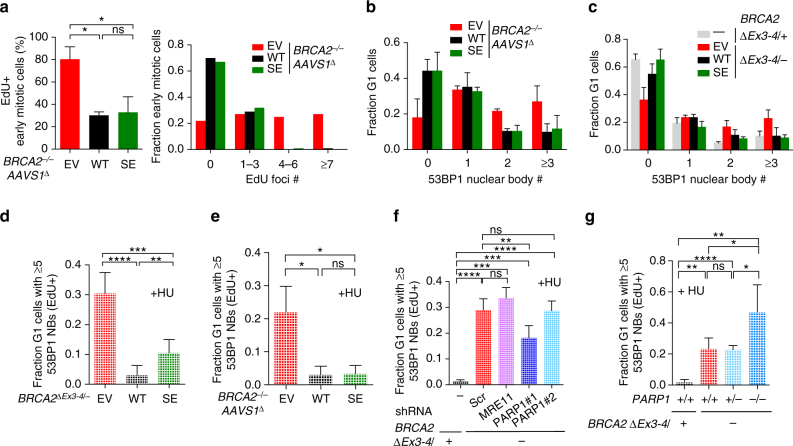
Fork protection is a minor replication stress suppression pathway. **a** Analysis of mitotic DNA synthesis in *BRCA2* ^*–/–*^ *AAVS1* ^*∆*^ cells complemented by BRCA2 WT or BRCA2 SE. Cells were released for 22 h from serum starvation to increase mitotic cells, incubated with EdU 1 h, and then analyzed for mitotic DNA synthesis. Early mitotic cells, defined as being in prophase, prometaphase, or metaphase, were analyzed for EdU foci that co-localize with FANCD2 foci pairs. The percent early mitotic cells containing EdU foci (*left*, *n* = 3) or EdU foci distribution in these cells (*right*, pooled results of three independent experiments) was quantified. **b**, **c** Spontaneous 53BP1 nuclear body analysis in G1 in both *BRCA2* ^*∆Ex3-4/–*^ cells (**b**) and *BRCA2* ^*–/–*^ *AAVS1* ^*∆*^ cells (**c**) complemented by BRCA2 WT or BRCA2 SE. *n* ≥ 3. **d**–**g** HU-induced 53BP1 nuclear body formation analysis, as in Fig. 4d, using complemented *BRCA2* ^*∆Ex3-4/–*^ cells (**d**) or *BRCA2* ^*–/*–^ *AAVS1* ^*∆*^ cells (**e**), stable MRE11 or PARP1 knockdown *BRCA2* ^*∆Ex3-4/*–^ cells (**f**), and *BRCA2* ^*∆Ex3-4/*–^ cells with the indicated *PARP1* genotype (**g**). *n* ≥ 3. *Error bars* s.d. ns, not significant; *p < 0.05; **p < 0.01; ***p < 0.001; ****p < 0.0001 (unpaired two-tailed *t*-test)

We asked whether fork protection or HR has a more critical role in suppressing replication stress. *BRCA2* ^*−/−*^ *AAVS1* ^*∆*^ cells expressing the BRCA2 SE protein, which is specifically impaired in fork protection, had similarly low levels of mitotic DNA synthesis as cells expressing BRCA2 WT (Fig. 6a). BRCA2 SE complemented cell lines also showed few spontaneous and HU-induced G1 53BP1 nuclear bodies, similar to BRCA2 WT cells (Fig. 6b–e). By contrast, *BRCA2* ^*∆Ex3-4/−*^ cells in which replication fork protection, but not HR, is restored through MRE11- or PARP1-deficiency showed high levels of HU-induced G1 53BP1 nuclear bodies (Fig. 6f, g). Altogether, these results imply that protection of stalled replication forks does not play a major role in suppressing DNA under replication and replication stress, as marked by G1 53BP1 nuclear bodies.

Thus far, our results show a correlation between HR proficiency, suppression of DNA under replication/53BP1 nuclear body formation, and cell viability: BRCA2 SE-expressing cells are at least partially competent in all aspects, whereas cells with combined BRCA2 and PARP1 deficiency are impaired in all aspects. Furthermore, a cross comparison of the effects of BRCA2 SE expression in the two conditional systems also reveals a correlation between HR activity and cell viability, as BRCA2 SE expression in *BRCA2* ^*−/−*^ *AAVS1* ^*∆*^ cells more completely restored both compared to in *BRCA2* ^*∆Ex3-4/−*^ cells (compare Supplementary Fig. 6d with Fig. 2h and Supplementary Fig. 6e, f with Fig. 2i).

**Fig. 7:**
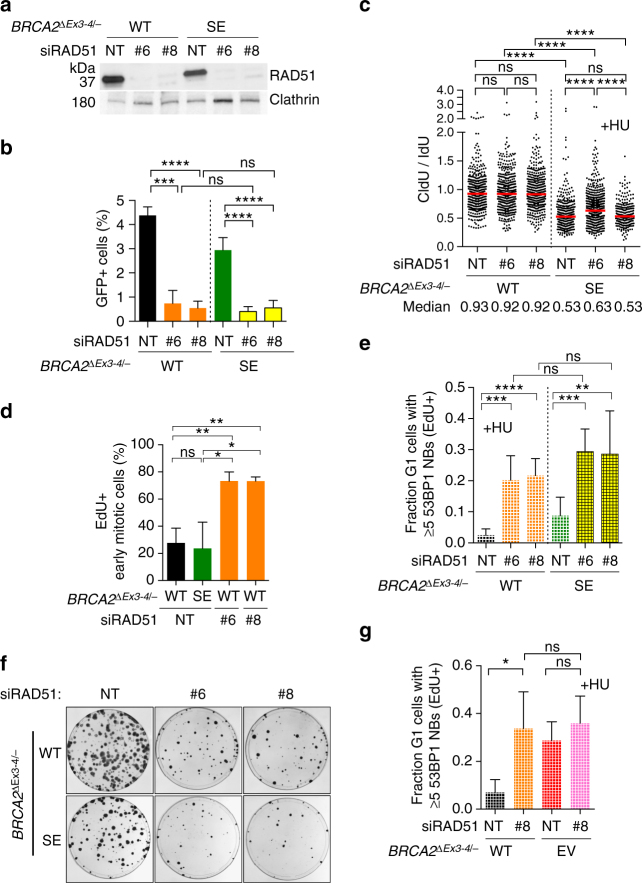
HR proficiency is associated with replication stress suppression and cell viability. **a** Western blot showing RAD51 knockdown in *BRCA2* ^*∆Ex3-4/*–^ cells stably expressing BRCA2 WT or BRCA2 SE. NT, non targeting siRNA. **b** HR analysis. Cells expressing RAD51 siRNAs were infected with I-SceI-expressing lentivirus and the percent GFP+ cells was analyzed by an unpaired two-tailed *t*-test. *n* ≥ 3. **c** Cells expressing RAD51 siRNAs were analyzed for fork protection in the presence of HU. Median CldU/IdU tract length ratios are indicated in the graph (*red bars*). *Graphs* represent the pooled results of >300 fibers per genotype from at least three independent experiments, analyzed by a two-tailed Mann–Whitney test. **d** Cells expressing RAD51 siRNAs were released for 22 h from serum starvation to increase mitotic cells, incubated with EdU 1 h, and then analyzed for mitotic DNA synthesis. Early mitotic cells, defined as being in prophase, prometaphase, or metaphase, were analyzed for EdU foci that co-localize with FANCD2 foci pairs. The percent early mitotic cells containing EdU foci were analyzed by an unpaired two-tailed *t*-test. *n* = 3. **e** HU-induced 53BP1 nuclear body formation analysis, as in Fig. 4d, using cells expressing RAD51 siRNAs. Statistical analysis was by an unpaired two-tailed *t*-test. *n* ≥ 4. **f** RAD51 depletion in *BRCA2* ^*∆Ex3-4/*–^ cells stably expressing BRCA2 WT or BRCA2 SE leads to a reduction in clonogenic survival. **g** HU-induced 53BP1 nuclear body formation analysis, as in Fig. 4d, using cells transfected with RAD51 siRNAs. Statistical analysis was by an unpaired two-tailed *t*-test. *n* = 4. *Error bars* s.d. ns, not significant; *p < 0.05; **p < 0.01; ***p < 0.001; ****p < 0.0001

To further investigate the importance of HR, we transiently depleted RAD51 (Fig. 7a, Supplementary Fig. 11a), the key strand exchange protein, which acts immediately downstream of BRCA2 in HR^3^. Cells showed a dramatic reduction in HR when RAD51 was depleted (Fig. 7b, Supplementary Fig. 11b), as expected. RAD51 depletion also causes a profound defect in repairing cisplatin-induced DNA damage, another hallmark of HR deficiency, to a similar extent as BRCA2-deficient cells (Supplementary Fig. 12a). However, protection of stalled replication forks was not adversely affected by RAD51 depletion (Fig. 7c, Supplementary Fig. 11c), consistent with recent observations^40^ (further examined below).

Based on these results, RAD51 depletion allowed us to investigate the consequences of disrupted HR independently of fork protection defects. Remarkably, RAD51 depletion in WT-complemented *BRCA2* ^*∆Ex3-4/−*^ cells substantially induced mitotic DNA synthesis (Fig. 7d, Supplementary Fig. 12b). After HU treatment, these cells also displayed markedly elevated levels of G1 53BP1 nuclear bodies (Fig. 7e). Concomitantly, cell survival was severely compromised in RAD51-depleted cells (Fig. 7f, Supplementary Fig. 11d). Thus, RAD51 depletion, with the consequent HR deficiency but adequate fork protection, is sufficient to cause replication stress associated with cell lethality. BRCA2 SE-complemented cells showed a small further decrease in colony formation upon RAD51 depletion compared to BRCA2 WT-complemented cells, although no further reduction in HR (Fig. 7e, f, Supplementary Fig. 11d), suggesting a compensatory role for fork protection in the absence of HR, although this warrants further investigation.

While consistent with recent observations^40^, the above result that RAD51 depletion does not cause nascent strand degradation is surprising given our previous findings using other approaches that have implicated RAD51 in fork protection^5, 6^. Interestingly, RAD51 depletion in *BRCA2* ^*∆Ex3-4/−*^ cells led to a partial restoration of fork protection (Supplementary Fig. 12c, d). Thus, while being critical for fork protection, RAD51 is also involved in a BRCA2-independent process that is upstream of nascent strand degradation, such that the overall outcome of RAD51 depletion does not affect replication fork stability. Importantly, RAD51 and BRCA2 co-deficiency did not further elevate 53BP1 nuclear bodies (Fig. 7g), indicating that RAD51 and BRCA2 function in the same pathway, which rules out that this putative RAD51-specific process plays a role in suppressing replication stress.

We also tested the possible involvement of fork restart. No detectible defects in resuming replication at stalled forks were observed in cells lacking RAD51, BRCA2, or both (Supplementary Fig. 12e), unlike a previous report using a tumor cell line with different treatment protocols^41^. Overall, our results suggest that HR is the primary pathway associated with the ability to suppress replication stress and support cell viability, while replication fork protection plays a minor, possibly compensatory role when HR activity is compromised.

### BRCA2 suppresses single-stranded DNA lesions in G2

**Fig. 8:**
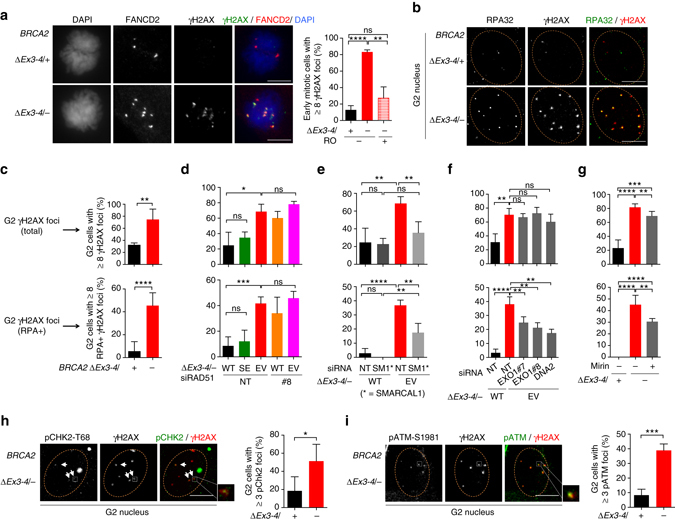
BRCA2-deficient cells accumulate single-stranded DNA lesions in G2. **a** γH2AX foci analysis in early mitotic cells. Cells were untreated, or treated with RO-3306 (10 µM, 24 h) to delay mitotic entry, and released for 1 h before analysis of γH2AX and FANCD2 foci pairs in early mitotic cells. Representative images are shown (*left*). Analysis is by an unpaired two-tailed *t*-test. *n* = 3. *Scale bars* 10 µm. **b**–**g** Cells treated with the indicated siRNAs or mirin (50 µM, 5 h) were incubated with EdU for 30 min and then analyzed for γH2AX and RPA foci in G2 cells (EdU–, 2N DNA content). Representative deconvolved images are shown (**b**). Quantification of γH2AX foci (*top*, **c**–**g**) and RPA+ γH2AX foci (*bottom*, **c**–**g**) for BRCA2-deficient cells (**c**), cells transfected with siRNAs (**d**, RAD51; **e** SMARCAL1; **f** EXO1 and DNA2), and cells treated with mirin (**g**) are shown. *n* ≥ 3. *Scale bars* 10 µm. **h**, **i** Cells were incubated with EdU as in **b** before analysis of γH2AX and pCHK2-T68 (**h**) or pATM-S1981 (**i**) foci in G2 cells. Representative deconvolved images with magnified *inset* highlighting foci co-localization are shown (*left* in each panel). *n* ≥ 3. *Scale bars* 10 µm. *Error bars* s.d. ns, not significant; *p < 0.05; **p < 0.01; ***p < 0.001; ****p < 0.0001 (unpaired two-tailed *t*-test)

To further explore the mechanisms by which BRCA2 prevents DNA under replication, we tested the hypothesis that unrepaired DNA damage in HR-deficient cells impedes timely replication completion. Early mitotic *BRCA2* ^*∆Ex3-4/−*^ cells displayed a dramatically increased level of γH2AX, with ~80% of cells containing  ≥8 γH2AX foci (Fig. 8a). Sites of under-replicated DNA, marked by FANCD2 foci pairs, typically co-occurred with these γH2AX foci, although not all γH2AX foci were marked by FANCD2. Importantly, these early mitotic DNA damage sites were diminished by delaying mitotic entry, implying that pre-mitotic DNA damage impedes replication completion.

We, therefore, assayed for possible lesions prior to mitosis in G2 phase. γH2AX foci were substantially induced in BRCA2-deficient cells in G2 and, remarkably, ssDNA, as indicated by RPA foci, was particularly enriched in these lesions (Fig. 8b, c). The ssDNA lesions were prevalent, although not all γH2AX-marked sites contained RPA foci (Fig. 8b). We considered the possibility that replication fork degradation is the cause of the observed ssDNA damage. However, BRCA2 SE expression in BRCA2-deficent cells effectively suppressed these G2 DNA lesions, despite impaired fork protection (Fig. 8d, Supplementary Fig. 13a). Conversely, RAD51 depletion, with HR but not fork protection deficiency, phenocopied BRCA2 deficiency in inducing ssDNA damage in G2, which was not further exacerbated by combined RAD51 and BRCA2 inactivation (Fig. 8d). These results are consistent with the notion that BRCA2 functions through RAD51-mediated HR to prevent DNA damage accumulation in G2.

Next, we investigated the mechanisms by which G2 DNA damage is generated. Replication fork reversal is considered to be a general response to different types of replication stress^42^ and reversed forks are susceptible to breakage^43, 44^. Indeed, depleting the fork remodeling protein SMARCAL1^43, 45, 46^ markedly reduced both overall and RPA+ G2 γH2AX foci produced in *BRCA2* ^*∆Ex3-4/−*^ cells (Fig. 8e, Supplementary Fig. 13b). Thus, although it remains to be directly tested whether BRCA2 loss itself affects fork reversal, our observations raise the possibility that fork reversal contributes to the formation of G2 lesions that arise in BRCA2-deficient cells. Reversed forks are subject to direct processing, a DNA2-specific function that is not shared by MRE11 or EXO1^40^. These four-way structures can also be cleaved by structure-specific endonucleases to produce DSBs^43, 44^, which can then undergo classical DNA end resection by nucleases, including MRE11, EXO1, and DNA2. Given the residual RPA+ γH2AX foci from SMARCAL1-depleted cells, it is also possible that lesions can arise independently of fork remodeling. To test the involvement of the resection enzymes in ssDNA formation, we transiently depleted DNA2 or EXO1 (Supplementary Fig. 13c, d) or inhibited MRE11 by mirin. Disruption of each individual resection enzyme in BRCA2-deficient cells led to a considerable reduction of RPA foci without markedly affecting the overall γH2AX level in G2 phase (Fig. 8f, g). Thus, although direct processing of reversed forks may play some role, the contribution of MRE11 and EXO1, in addition to DNA2, in producing ssDNA is consistent with end resection of DNA breaks. Indeed, we detected ATM pathway activation, manifested by foci of phosphorylated ATM and CHK2 at the damage sites (Fig. 8h, i), indicating break formation. Altogether, our results suggest that fork reversal, DNA breakage, and hyper-resection contribute substantially to the lesions that accumulate in G2 phase upon HR deficiency and that these persistent intermediates compromise the timely completion of replication.

## Discussion

BRCA2 germline mutation predisposes to breast and ovarian cancer. Seemingly paradoxically, however, BRCA2 deficiency results in inviability both during mouse embryo development and in mouse cells themselves^12–15^. How the cell lethality is triggered in normal cells and bypassed during tumor formation remains unclear. Here, using a BRCA2 conditional system in a non-transformed human mammary epithelial cell line, we show that BRCA2 deficiency induces replication stress, resulting in ssDNA lesions in G2, failure to complete DNA replication and concomitant 53BP1 nuclear body formation in the subsequent G1 phase, to ultimately lead to p53-dependent G1 arrest and cellular senescence. Importantly, suppression of replication stress and support of cell viability mainly associate with the HR function of BRCA2.

**Fig. 9:**
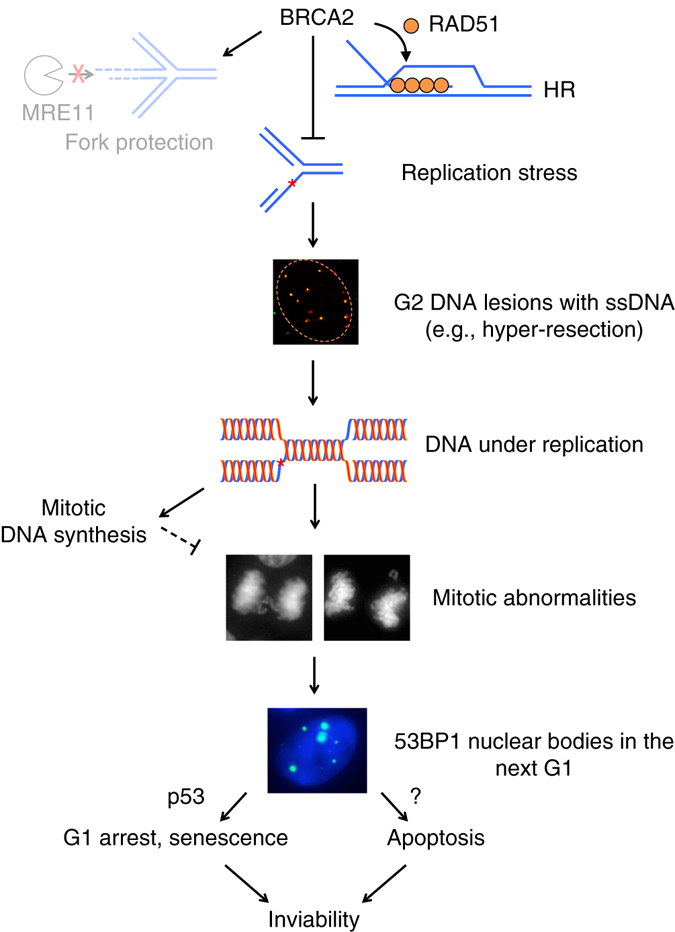
Model for replication stress and its aftermath in the absence of BRCA2. BRCA2 suppresses replication stress and DNA under replication in non-transformed cells primarily through RAD51-mediated HR repair of DNA damage. Protection of stalled replication forks from MRE11-mediated degradation plays a minor role. Upon BRCA2 deficiency, single-stranded DNA lesions accumulate in G2, generated, in part, by fork reversal (not shown) and hyper-resection. Unrepaired DNA damage perturbs the timely completion of DNA replication, leading to under replication. During early mitosis, the compensatory mitotic DNA synthesis pathway is insufficient, such that these unresolved DNA structures lead to anaphase abnormalities and formation of 53BP1 nuclear bodies in the next G1 phase. G1 arrest and cellular senescence mediated by p53 and p53-independent apoptosis are in turn triggered to ultimately result in cell inviability

To dissect the mechanism by which BRCA2 functions to prevent replication stress and support cell viability, we generated multiple, complementary separation-of-function systems to distinguish the roles of HR and fork protection: HR was specifically disrupted by RAD51 depletion in wild-type cells, while fork protection was specifically impaired by BRCA2 SE expression or restored by MRE11 or PARP1 deficiency in BRCA2-deficient cells. Taken together, these systems demonstrate that protection of stalled forks plays a minor role in suppressing replication stress and promoting cell proliferation; rather, they support the conclusion that BRCA2 primarily functions through HR in these processes (Fig. 9). We cannot formally exclude possible contributions of some as yet unknown BRCA2-RAD51-mediated process that is separable from strand invasion. However, thus far, the various genetic systems tested, with the potentially confounding pathways excluded (i.e., fork protection and restart), are consistent with a role of HR in preventing DNA under replication and its aftermath. This model can explain the viability of mice and humans whose cells show reasonable levels of HR but are nonetheless deficient in fork protection, for example, those with Fanconi anemia or *Brca2* hypomorphic mutation^5, 6, 47–49^.

Our data provide mechanistic insight into how BRCA2 suppresses replication stress and under replication. First, BRCA2 serves to repair replication-associated DNA damage such as DSBs through HR (Fig. 9), as it does in other contexts. Supporting this, we observe that BRCA2 deficiency leads to spontaneous DNA damage originating in S/G2 phases that persists into mitosis. In particular, at least a fraction of the G2 damage is characterized as hyper-resected DNA arising from activities of resection enzymes that are known to generate intermediates for strand invasion during HR, although other resection-independent mechanisms could also be involved, for example, ssDNA gap formed behind the fork that has been seen upon RAD51 impairment^42, 50^. These lesions, if left unrepaired or inefficiently repaired, can in turn impede completion of DNA replication. For example, BRCA2 or RAD51 deficiency biases stalled fork-induced recombination towards long-tract gene conversion^51^; more repair synthesis than during canonical HR may delay the completion of replication. Second, BRCA2 may facilitate DNA replication completion in a DSB-independent manner. In particular, RAD51-mediated fork reversal^42^, followed by BRCA2-promoted strand invasion by RAD51, may allow lesion bypass without DNA breakage. Lastly, through its HR function, BRCA2 may additionally be involved in the following S phase to promote the resolution of lesions marked by 53BP1 nuclear bodies.

The description of aberrant mitotic structures in BRCA2-deficient cells is not entirely unprecedented but has often been explained by mitotic-specific functions of BRCA2^52–55^. While we do not rule out the possibility of a mitotic-specific function of BRCA2, the restoration of mitotic integrity in BRCA2-deficient cells by a pre-mitotic treatment—delayed mitotic entry—strongly suggests that the underlying lesions occur in the preceding cell cycle phases (i.e., DNA under replication in S phase). Moreover, the aberrant mitotic structures are associated with sites of DNA under replication. Delaying mitotic entry, as well as prolonging prometaphase, the stage when compensatory mitotic DNA synthesis occurs, also rescues G1 abnormalities (i.e., 53BP1 nuclear body formation), further supporting the notion that lack of replication completion causes mitotic and G1 abnormalities upon BRCA2 deficiency.

At first glance, the finding of a minor role for fork protection, specifically, protection of nascent strands, is surprising considering its recently reported critical role in supporting viability of mouse ES cells and conferring chemoresistance to tumor cells^8, 9^. We envision diverse pathway choices to maintain genomic integrity and/or support viability in different biological contexts. Given our results that the p53 pathway impedes cell survival, the threshold to survive BRCA2 loss, and HR loss more generally, may be lower in mouse ES cells due to their compromised p53-mediated G1/S checkpoint^56^; thus, even with HR deficiency, reducing DNA damage by protecting stalled forks may be sufficient for cell survival^8^. However, fork protection is not sufficient to fully support embryonic development of *Brca2*-deficent mice^8^ during which differentiation and the accompanying restoration of G1/S checkpoint function occur. Similarly, having survived the crisis of BRCA2 loss by p53 mutation and/or other means, tumor cells may be able to bypass the requirement for HR, such that restoration of fork protection is then sufficient to deal with replication stress from agents like olaparib and cisplatin, as recently observed^9^. However, it is important to note that HR is restored through reversion mutations in a substantial fraction of therapy-resistant human tumors^57, 58^, such that HR reactivation cannot be underestimated as a major mechanism of therapy resistance.

In an effort to model normal mammary tissue, we used a non-transformed human mammary epithelial cell line, providing evidence that HR is more critical than fork protection for genome integrity and cell viability. We cannot rule out that the specific genetic background of MCF10A cells^59^ could influence our findings, such that experiments in other normal mammary contexts will be required to formally test the generalizability of our findings. Nevertheless, a high reliance on HR in mammary cells is supported by recent in vivo studies, showing particularly robust HR in mammary tissue compared to other tissues^48^. Collectively, these previous and our current studies using various systems lead us to propose the complexity of the contribution that these genome integrity maintenance pathways (i.e., HR and fork protection) make in different biological systems.

Our observation that BRCA2 deficiency induces replication stress adds a new dimension to a growing literature that replication stress is a key feature of precancerous lesions induced by oncogenes^60^. Unanticipated consequences of the replication stress induced by BRCA2 loss are mitotic abnormalities leading to G1 arrest and 53BP1 nuclear body formation, which may be exploitable as a diagnostic biomarker for BRCA2 status in carriers. Whether the sequelae of replication stress that we observe with loss of BRCA2, in particular 53BP1 body formation, will be found more generally such as in oncogene-induced precancerous lesions will be important to determine.

Our studies also have implications for cancer therapy. Agents found to enhance 53BP1 nuclear body formation may further sensitize BRCA2-deficient cancer cells to therapy. DNA under replication could also potentially be exploited as an Achilles heel to treat BRCA2-deficient cancers by targeting components in the mitotic DNA synthesis pathway. While a previous study demonstrated the activation of mitotic DNA synthesis in the presence of aphidicolin^61^, our finding here that mitotic DNA synthesis is activated upon BRCA2 loss even in the absence of exogenous replication stress suggests that it is more relied upon and, therefore, a promising target for intervention in BRCA2-deficient tumors.

## Methods

### MCF10A cell culture and drug treatment

MCF10A cells, obtained from ATCC through B.H. Park (Johns Hopkins University School of Medicine)^62^ and tested negative for mycoplasma contamination, were grown in DME-HG/F-12 supplemented with 5% horse serum, 20 ng ml^−1^ epidermal growth factor, 0.5 mg ml^−1^ hydrocortisone, 100 ng ml^−1^ cholera toxin, 10 µg ml^−1^ insulin, and 1% penicillin–streptomycin. IdU (50 µM; I7125, Sigma), CldU (50 µM; C6891, Sigma), hydroxyurea (4 mM; H8627, Sigma), cisplatin (5 µM; 479306, Sigma), olaparib (5 µM, MSKCC Organic Chemistry Core Facility), mirin (50 µM, MSKCC Organic Chemistry Core Facility), RO-3306 (10 µM; SML0569, Sigma), and nocodazole (100 ng ml^−1^; M1404, Sigma) were used at the indicated concentrations.

### Plasmid construction

For *BRCA2* Ex3-4flox donor plasmid (Ex3-4 fl-Hyg) construction, sequences containing loxP, FRT sites, and the SA-2A module were synthesized and cloned together with a hygromycin-resistance gene between *Spe*I/*Sal*I sites of the pBluescript II SK+ backbone. The *BRCA2* left homology arm, right homology arm, and exon 3 and 4 region were amplified from genomic DNA from MCF10A cells and sequentially cloned into the above vector with the PAM sequence of the sgRNA recognition site removed. The *BRCA2* “–” allele donor plasmid was constructed by replacing the left and right homology arms in an existing hygromycin targeting plasmid^63^; a blasticidin-resistance cassette replaced the hygromycin cassette where indicated. The TrBRC5-Cter DNA sequence was amplified in two parts from a previously described plasmid^21^ and stepwise cloned between *Eco*RI/*Sal*I sites of the *AAVS1* donor plasmid (a gift from Dr Dirk Hockemeyer). Homology arms for all targeting vector are designed to be 700–900 bp in length.

*BRCA2* expression vectors were generated by cloning the full-length *BRCA2* sequence from pcDNA3-BRCA2^64^ between *Xho*I/*Not*I sites of the PiggyBac transposon plasmid (a gift from Drs. David Allis and Ping Chi) together with a 3XFLAG tag fused to the N terminus. The *BRCA2* S3291E mutation was introduced by replacing the DNA fragment between *Age*I/*Not*I sites in wild-type *BRCA2* with the corresponding region from the FE-BRCA2-TR2 plasmid^65^.

The lentiviral vector that expresses I-SceI endonuclease for the HR assay was generated by replacing the fragment between *Spe*I/*Sal*I sites of the pCDH-CMV-MCS-EF1-copGFP vector (CD511B-1, System Biosciences) with the CAGGS-I-SceI fragment from the pCBASce plasmid^21^. DNA sequences of all constructs were confirmed by Sanger sequencing. The *BRCA2* Ex3-4flox donor plasmid contained a few polymorphisms in the 6-kb intron 3 that did not affect *BRCA2* expression.

sgRNAs were cloned into a non-viral backbone (Addgene plasmid # 41824)^66^ or a lentiGuide-puro backbone (Addgene plasmid # 52963)^67^, as described. Short hairpin RNA (shRNA) expression vectors were generated by cloning the target sequences between *Age*I/*Eco*RI sites of the pLKO.1-NeoR backbone, which was modified from the original pLKO.1 vector (Addgene plasmid # 1864)^68^ by swapping the puromycin-resistance gene with the neomycin-resistance gene.

### Lentiviral transduction

Lentivirus was produced by standard methods. Briefly, HEK293T cells at 80% confluence were co-transfected with a lentiviral vector, VSV-G expression plasmid and psPAX2 by Lipofectamine 2000 (11668027, Thermo Fisher Scientific) following the manufacturer’s instructions. The envelope and packaging vectors were gifts from Dr Ping Chi. Supernatants containing virus were collected and 0.45-µm filtered 48 and 72 h after transfection. Infections of MCF10A cells were performed in the presence of 8 µg ml^−1^ polybrene (TR-1003-G, EMD Millipore).

### *BRCA2* gene targeting and complementation

To achieve gene targeting, cells were co-transfected with a donor plasmid and vectors expressing either AAVS1 TALENs (for AAVS1 targeting)^69^ or Cas9-sgRNA (for other targeting purposes). Wild-type Cas9 (Addgene plasmid # 41815)^66^ or paired nickases (Cas9 H840A)^70, 71^ were used. Transfections were performed either by electroporation (Gene Pulser II, Bio-Rad; 350 V, 1000 µF) or nucleofection (Amaxa® Nucleofector® II, Lonza; program X-005). Cells were treated with drugs for selection 2 or 3 days post transfection depending on the selectable marker: hygromycin (100 µg ml^−1^), G418 (0.2 mg ml^−1^), or blasticidin (5.0 µg ml^−1^). The hygromycin-resistance gene cassette from *BRCA2* ^*fl-Hyg/+*^ cells was removed by transfection of the Flpo plasmid (a gift from Dr Prasad Jallepalli); colonies were analyzed to identify *BRCA2* ^*fl/+*^ clones. *BRCA2* ^*fl/−*^ cells were subsequently generated by introducing the *BRCA2* “–” allele donor plasmid into *BRCA2* ^*fl/+*^ cells.

sgRNA target sequences:

BRCA2-In2 (Ex3-4 fl-Hyg allele targeting): CTATAGATTCGCAAGAGAA

BRCA2-Ex2-9 (BRCA2 “–” allele targeting): AGACTTATTTACCAAGCAT

BRCA2-Ex2-6 (BRCA2 “–” allele targeting, used together with sgBRCA2-Ex2-9 for paired nickase strategy^70^): GCCTCTCTTTGGATCCAAT

Gene targeting was confirmed by Southern blotting. Genomic DNA was digested with the indicated restriction enzymes overnight at 37 °C and then electrophoresed on a 0.8% agarose gel and transferred to a charged nylon membrane (NEF987001PK, PerkinElmer). Probes were radiolabeled with [α-^32^P]-dATP using a random primer labeling kit (Agilent) and hybridized with the membrane overnight at 67 °C. The membrane was then washed three times with saline-sodium citrate buffer containing 0.1% SDS and developed.

For stable *BRCA2* complementation, the PiggyBac plasmid was co-transfected with a transposase expression plasmid (a gift from Drs. David Allis and Ping Chi) into cells by nucleofection and G418 (0.2 mg ml^−1^) selection was applied 48 h later to obtain G418-resistant cell pools. For BRCA2 expression in *BRCA2* ^*−/−*^ *AAVS1* ^*∆*^ cells, the neomycin-resistance gene at the targeted *AAVS1* locus was first inactivated by CRPSPR-Cas9 followed by screening for G418-sensitive clones. sgRNA target in Neo: GCTGACAGCCGGAACACGG

For *BRCA2* conditional deletion, cells were infected with purified adeno-Cre (Ad5-CMV-Cre, Baylor College of Medicine Vector Development Laboratory) and applied at a multiplicity of infection of 1000 in the presence of 1.2% Genejammer (204130, Agilent). Alternatively, cells were infected with a lentivirus that expresses a self-deleting Cre^20^. Both methods generated similar results. Downstream assays were performed using cell pools at least 72 h after infection, except the S/G2 entry experiments (Fig. 4a, Supplementary Fig. 9), which were performed 2 days after infection as indicated in the figures.

Genotyping PCRs to distinguish the Cre-excised (∆) from the unexcised (fl) allele were performed using genomic DNA prepared with the PureLink™ Genomic DNA Kit (K182002, Thermo Fisher Scientific) following the manufacturer’s instructions. Alternatively, cell pellets were resuspended in PCR grade water and heated at 100 °C for 5 min. The resulting extract was directly used for PCR. Oligo 1 (forward) and 2 (reverse) were used to detect the excised *∆Ex3-4* allele in *BRCA2* ^*∆Ex3-4/−*^. Oligos 1 (forward) and 3 (reverse) were used to detect the wild-type (+) or unexcised *fl* allele in *BRCA2* ^*fl+*^ and *BRCA2* ^*fl/−*^ cells. Oligo 4 (forward) and 5 (reverse) were used to detect the excised *∆* allele (yielding a 400 bp product) in *BRCA2* ^*−/−*^ *AAVS1* ^*∆*^ cells. Unexcised allele would yield a product that is too long (6.5 kb) to be amplified. Oligo 6 (forward) and 7 (reverse) were used to detect the unexcised *fl* allele (yielding a 460 bp product) in *BRCA2* ^*−/−*^ *AAVS1* ^*fl*^ cells. This PCR from excised allele is not productive due to removal of the Oligo 6 binding site by Cre.

Oligo 1: ACTTTTGTGAACTCTTGTTACACC

Oligo 2: GGTGTATGAAACAAACTCCCAC

Oligo 3: CTAAGATTTTAACACAGGTTTGCC

Oligo 4: ATTGTGCTGTCTCATCATTTTGGC

Oligo 5: CAGGAAATGGGGGTGTGTCAC

Oligo 6: TGTGGCACCAAATACGAAACACC

Oligo 7: ACAAATGTGGTATGGCTGATTATG

For RT-PCR, RNA was prepared using RNeasy Plus Mini Kit (74134, Qiagen) following the manufacturer’s instructions. Complementary DNA was then synthesized from RNA using SuperScript® III First-Strand Synthesis Kit (18080051, Thermo Fisher Scientific) following the manufacturer’s instructions. Oligo 8 (forward) and 9 (reverse) were used to amplify ex1–10 region from BRCA2 transcript.

Oligo 8: GAAGCGTGAGGGGACAGATTTG

Oligo 9: TACTTCATCTTCTAGGACATTTGG

### Gene knockout

p53 knockout cells were generated by co-transfection of vectors expressing Cas9 and an sgRNA for *TP53* (a gift from Dr Prasad Jallepalli) by nucleofection. Single colonies were picked and screened by western blotting. To knock out PARP1, a *BRCA2* ^*fl/−*^ clone that stably expresses Cas9 was first generated from viral infection of lentiCas9-Blast (Addgene plasmid # 52962)^67^. (Note these cells also stably express Cre-ERT2 from the pQCXIN backbone, a gift from Dr Prasad Jallepalli, although Cre remains inactive in all experiments in this study.) Cells were next nucleofected with a vector for an sgRNA for *PARP1* in a lentiGuide-puro backbone (Addgene plasmid # 52963)^67^. One day after transfection, cells were transiently selected with puromycin (1 µg ml^−1^) for 2 days before drug removal and being plated for growth as single colonies. Clones were screened based on restriction site loss at the Cas9 cleavage site and knockouts were confirmed by western blotting.

sgRNA target sequences:

p53: GGCAGCTACGGTTTCCGTC

PARP1: AACGTCAGGGTGCCGGA

### RNA interference

Stable knockdown cell lines were generated by infecting cells with viruses expressing the target shRNA, followed by continuous G418 (0.2 mg ml^−1^) selection.

shRNA target sequences:

MRE11^72^: GATGAGAACTCTTGGTTTAAC

PARP1-1 (TRCN0000356475): GGAGACCCAATAGGCTTAATC

PARP1-2 (TRCN0000338407): CTGATCCTTCAGCTAACATTA

Transient knockdown with Small interfering RNA (siRNAs) by Lipofectamine RNAiMax (13778075, Thermo Fisher Scientific), according to the manufacturer’s instructions, was performed 24 or 48 h after Cre infection. siRNAs were purchased as follows: RAD51 (Qiagen, siRAD51#6, SI02629837; #8, SI03061338), SMARCAL1 (SMARTpool, Dharmacon, M-013058-01-0005), EXO1 (Qiagen, siEXO1#7, SI02665138; #8, SI02665145), DNA2 (SMARTpool, Dharmacon, M-026431-01-0005). Cells were analyzed 48 h after transfection. A scrambled shRNA^68^ and a non-target siRNA (1027281, Qiagen) were used as negative controls.

### Cell proliferation and clonogenic survival assays

For proliferation assays, cells were counted, diluted and plated 3 days after Cre infection (passage 0). Two further rounds of cell counting and re-plating were performed (passage 1 and 2) at 3-day intervals. Cell numbers were normalized to the number of *BRCA2* ^*∆Ex3-4/+*^ cells at passage 0.

For clonogenic survival assays, 1000–2000 cells were seeded in a 10 cm plate and stained by Giemsa (620G-75, EMD Millipore) after methanol fixation 11 days later. Plating efficiency was calculated as the ratio of the amount of colonies formed to the total number of cells plated. All clonogenic assays from this study were performed in an unchallenged condition (i.e., no exogenous DNA damage applied).

### Cell senescence and apoptosis assays

Senescence-associated β-galactosidase staining was performed following manufacturer’s instructions (9860S, Cell Signaling Technology). The fraction of β-galactosidase+ cells was quantified using ImageJ software. Apoptotic cells were labeled using a FITC Annexin V kit (640905, BioLegend) according to the manufacturer’s instructions, followed by flow cytometry (Becton Dickinson FACScan) and quantification by FlowJo software.

### Cell cycle analysis

Cells were fixed in ice-cold 70% ethanol overnight, before being pelleted, resuspended, and then incubated in propidium iodide (PI) staining buffer (20 μg ml^−1^ PI, 0.2 mg ml^−1^ RNase A, 0.1% Triton-X, PBS) for 30 min at room temperature. Where indicated, cells were incubated with EdU for 30 min before harvest. In this case, EdU was detected using Click-iT® Plus EdU Alexa Fluor® 488 Flow Cytometry Assay Kit (C10632, Thermo Fisher Scientific) following the manufacturer’s instructions and 7-AAD (420403, BioLegend) was used in place of PI for DNA staining. Cell cycle distribution was analyzed by flow cytometry (Becton Dickinson FACScan) and FlowJo software.

### DNA fiber assay

DNA fiber assays were performed as previously described^5^. Briefly, cells were pulse labeled with 50 µM IdU and 50 µM CldU, untreated or treated with 4 mM HU, as indicated. 2000–4000 cells were lysed in lysis buffer (0.5% SDS, 200 mM Tris-HCl pH 7.4, 50 mM EDTA). DNA fibers were spread on microscope slides and fixed in methanol/acetic acid (3:1 by volume). DNA was denatured in 2.5 M HCl for 30 min, followed by 1 h blocking buffer (10% goat serum and 0.1% Triton-X in PBS). Slides were incubated with primary antibodies, anti-CldU (1:75; MA1-82088, Thermo Fisher Scientific) and anti-IdU (1:75; 347580, BD Biosciences) followed by secondary antibodies, anti-rat Alexa Fluor® 488 and anti-mouse Alexa Fluor® 594 (1:250, Thermo Fisher Scientific), for 1 h each in blocking buffer at room temperature. Slides were mounted in Prolong with DAPI (P36935, Thermo Fisher Scientific) before image acquisition under Axio2 microscope (Zeiss). Images were analyzed with FIJI (ImageJ) software.

### HR assay

All cell lines used are derived from an MCF10A cell clone that contains the DR-GFP reporter^22^ stably integrated as a single copy in the genome (a gift of Dr Elizabeth Kass). Cells were infected with I-SceI-expressing lentivirus and HR was measured by quantifying the fraction of GFP+ cells by flow cytometry (Becton Dickinson FACScan) 48 h after infection using FlowJo software.

### Immunofluorescence and microscopy

Cells were cultured on Nunc™ Lab-Tek™ II CC2™ Chamber Slides (12-565-1, Thermo Fisher Scientific) and fixed with 2% paraformaldehyde for 15 min and permeabilized in blocking buffer (0.1% Triton-X and 1% BSA in PBS) for 30 min at room temperature. To stain chromatin-bound RPA and MRE11, cells were pre-extracted (0.5% Triton-X, 1 mM EDTA, 30 mM sucrose in PBS) on ice for 5 min before fixation. Where indicated, EdU was detected using Click-iT® Plus EdU Alexa Fluor® 647 Imaging Kit (C10640, Thermo Fisher Scientific) following manufacturer’s instructions, except that CuSO_4_, Alexa Fluor® azide and reaction buffer additive were used at half of the instructed final concentrations. Cells were then incubated with primary antibodies followed by secondary antibodies diluted in blocking buffer for 1 h each with three PBS washes in between. Slides were mounted in Prolong with DAPI (P36935, Thermo Fisher Scientific) before image acquisition under Axio2 microscope (Zeiss). Where indicated, deconvolution was carried out with z stacks acquired with 0.2 µm spacing using enhanced ratio method, and projected based on maximum intensity on a DeltaVision Image Restoration System (GE Healthcare). Quantification of fluorescence signal intensity per nucleus with was performed with high-content image-based cytometry methods essentially as described^73^ using FIJI (ImageJ) and analyzed using Excel (Microsoft) softwares. Briefly, nucleus regions were segmented based on total DAPI intensity, and mean fluorescence intensities of other channels within each nucleus were quantified in FIJI and exported to Excel where data analysis was performed. Replicate experiments for γH2AX intensity quantification are shown in Supplementary Fig. 14.

Primary antibodies used were γH2AX (1:1000; 05-636, EMD Millipore; 1:500; 2577 S, Cell Signaling Technology), 53BP1 (1:1000; 612522, BD Biosciences), cyclin A (1:1000; sc-751, Santa Cruz Biotechnology), FANCD2 (1:500; NB 100-182, Novus Biologicals), MRE11 (1:1000; a gift from Dr John Petrini), PICH (1:500; H00054821-M01, Novus Biologicals), pATM-S1981 (1:1000; 200-301-400, Rockland), pCHK2-T68 (1:1000; 2661S, Cell Signaling Technology), RPA (1:1000; ab2175, Abcam; 1: 1000, 2208S, Cell Signaling Technology). Secondary antibodies used were anti-mouse Alexa Fluor® 488, anti-rat Alexa Fluor® 488, anti-mouse Alexa Fluor® 594, anti-mouse Alexa Fluor® 647, anti-rabbit Alexa Fluor® 568, and anti-rabbit Alexa Fluor® 594 (1:1000; Thermo Fisher Scientific).

### Serum starvation and mitotic cell analysis

For serum starvation, cells were cultured for 24 h in DME-HG/F-12 with 1% penicillin–streptomycin but no other additive. Cells were then released into regular culture media at the indicated time interval. To detect mitotic DNA synthesis, EdU was added to the media for another 1 h incubation before fixation and permeabilization as described in the immunofluorescence section. EdU was detected using Click-iT® Plus EdU Alexa Fluor® 488 Imaging Kit (C10637, Thermo Fisher Scientific) following manufacturer’s instructions, except that CuSO_4_, Alexa Fluor® azide and reaction buffer additive were used at half of the instructed final concentrations. Slides were mounted in Prolong with DAPI (P36935, Thermo Fisher Scientific). Mitotic cells were detected by microscopy. More than 80 mitotic cells from at least three independent experiments were scored.

### Subcellular fractionation and immunoprecipitation

Subcellular fractionation was performed using the Subcellular Protein Fractionation Kit (78840, Thermo Fisher Scientific) following the manufacturer’s instructions. For protein lysate preparation, cells were trypsinized and lysed with NETN lysis buffer (150 mM NaCl, 1 mM EDTA, 20 mM Tris pH 8.0, 0.5% NP40, 10% Glycerol) containing protease inhibitor (11836153001, Roche). For FLAG-IPs, EZview™ Red ANTI-FLAG M2 Affinity Gel (F2426, Sigma) was added to 0.5–2.0 mg protein lysate and incubated overnight at 4 °C. After extensive washes with 0.05% Tween 20 in PBS, proteins were eluted with SDS-PAGE sample buffer (B7703S, NEB).

### Western blotting

Equal amounts of protein samples (whole-cell lysate, subcellular fractions, or IP samples) were heated at 70 or 100 °C for 10 min, run on a precast Tris-acetate (for BRCA2 blots; EA03752BOX, Thermo Fisher Scientific) or Mini-PROTEAN® TGX™ protein gel (Bio-Rad) and then transferred to a nitrocellulose membrane (162-0145, Bio-Rad). The membrane was blocked in 5% non-fat dry milk in PBST (PBS with 0.05% Tween-20) and incubated overnight with primary antibodies at 4 °C, followed by incubation with secondary antibodies for 1 h at room temperature. Uncropped images of western blots are shown in Supplementary Fig. 15.

Primary antibodies used were BRCA2 (1:300; OP95, EMD Millipore), clathrin (1:3000; 610499, BD Biosciences), DNA2 (1:500; ab96488, Abcam), EXO1 (1:1000; A302-640A-T, Bethyl Laboratories), FLAG (1:1000; A8592, Sigma), MRE11 (1:5000; a gift from Dr John Petrini), PARP1 (1:1000; sc-7150, Santa Cruz Biotechnology), p53 (1:1000; sc-98, Santa Cruz Biotechnology), p21 (1:1000; sc-6246, Santa Cruz Biotechnology), HDAC2 (1:2000; 2540S, Cell Signaling Technology), SMARCAL1 (1:500; sc-376377, Santa Cruz Biotechnology), tubulin (1:10,000; T9026, Sigma), RAD51 (1:2000; PC130, EMD Millipore), histone H3 (1:2000; 9715, Cell Signaling Technology). Secondary antibodies used were peroxidase-linked anti-mouse or anti-rabbit IgG (1:10,000; GE Healthcare).

### Statistical analysis

Statistical analysis was performed using Prism software. *p*-values for fork protection, γH2AX, MRE11 nuclear intensity and FANCD2 foci pair quantification were determined using a two-tailed Mann–Whitney test. The remaining data were analyzed by an unpaired two-tailed *t*-test. Statistical tests were justified appropriate for every figure (see legends) and the variance between groups was usually similar. No statistical methods or criteria were used to estimate sample size or to include or exclude samples. For DNA fiber analysis, investigators were blinded in most experiments to the group allocation; for other experiments, investigators were not blinded. *p*-values of <0.05 are considered statistically significant and are indicated with asterisks as follows: *p < 0.05; **p < 0.01; ***p < 0.001; ****p < 0.0001.

### Data availability

All relevant data are available from the authors on request.

## Electronic supplementary material

Supplementary Information
