# Supplementary material for: BRCA2 suppresses replication stress-induced mitotic and G1 abnormalities through homologous recombination
Source: Nat Commun. 2017 Sep 13;8:525. doi: 10.1038/s41467-017-00634-0 (PMC5597640; doi:10.1038/s41467-017-00634-0)
Supplement: Supplementary file 1 — Supplementary Information [file 41467_2017_634_MOESM1_ESM.pdf]

Description of Supplementary Files

File Name: Supplementary Information
Description: Supplementary Figures

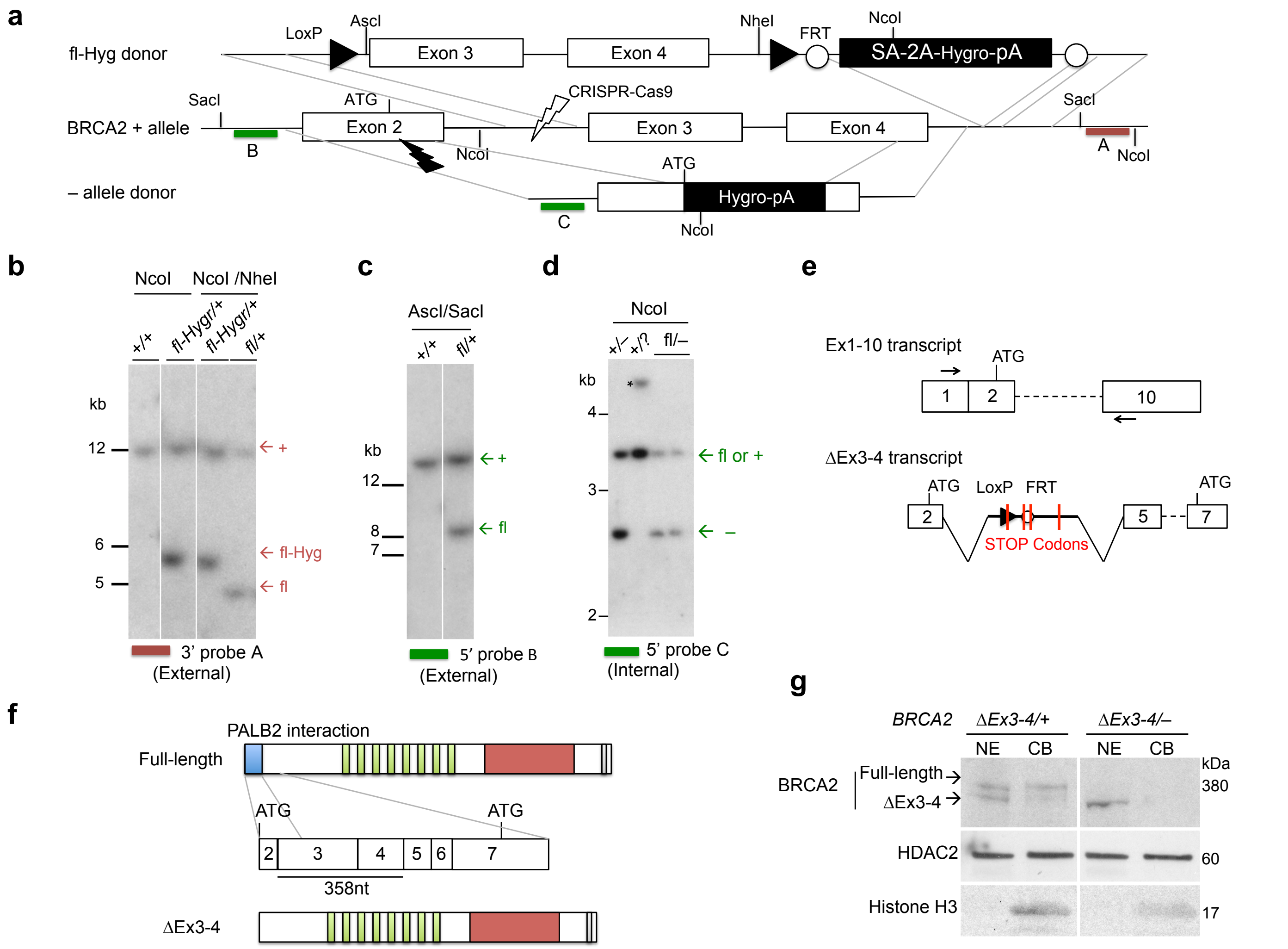

Supplementary Figure 1. Generation of *BRCA2*$^{fl/–}$ and *BRCA2*$^{\Delta Ex3\text{-}4/–}$ cells

a. Schematic for generation of *BRCA2*$^{fl/–}$ cells by CRISPR-Cas9-mediated gene targeting. Donor fragments for generating the BRCA2 exon 3-4 floxed allele prior to Flp recombination (fl-Hyg) and the null (-) allele are shown. Restriction digestion sites for Southern blotting are indicated. SA-2A, splice acceptor followed by 2A self-cleaving peptide sequence; lightning symbols, CRISPR-Cas9 recognition sites; colored bold lines, probes used for Southern blotting.

b-d. Southern blots to confirm targeting of MCF10A cells. The correct bands for the various alleles are indicated by the arrows. Asterisk indicates an unexpected band with the corresponding allele designated with a question mark; this clone was not further interrogated.

e. RT-PCR strategy to detect splicing from the Δ*Ex3-4* transcript. Using primers that hybridize to BRCA2 exons 1 and 10, a product is detected that contains a portion of intron 2 spliced to intron 4 with several stop codons that are in frame with the normal BRCA2 start codon. The next in-frame ATG codon in exon 7 is indicated.

f. Schematic showing domains of BRCA2 protein expressed from + (full-length) and that predicted from the Δ*Ex3-4* allele (ΔEx3-4) if translation begins in exon 7. Additional ATGs downstream can also be used to give rise to a similar peptide.

g. Western blot of BRCA2 after subcellular protein fractionation, showing that the ΔEx3-4 peptide is defective in binding to chromatin (NE, soluble nuclear extract; CB, chromatin-bound fraction). The BRCA2 antibody Ab-1 detects BRCA2 amino acids 1651-1821.

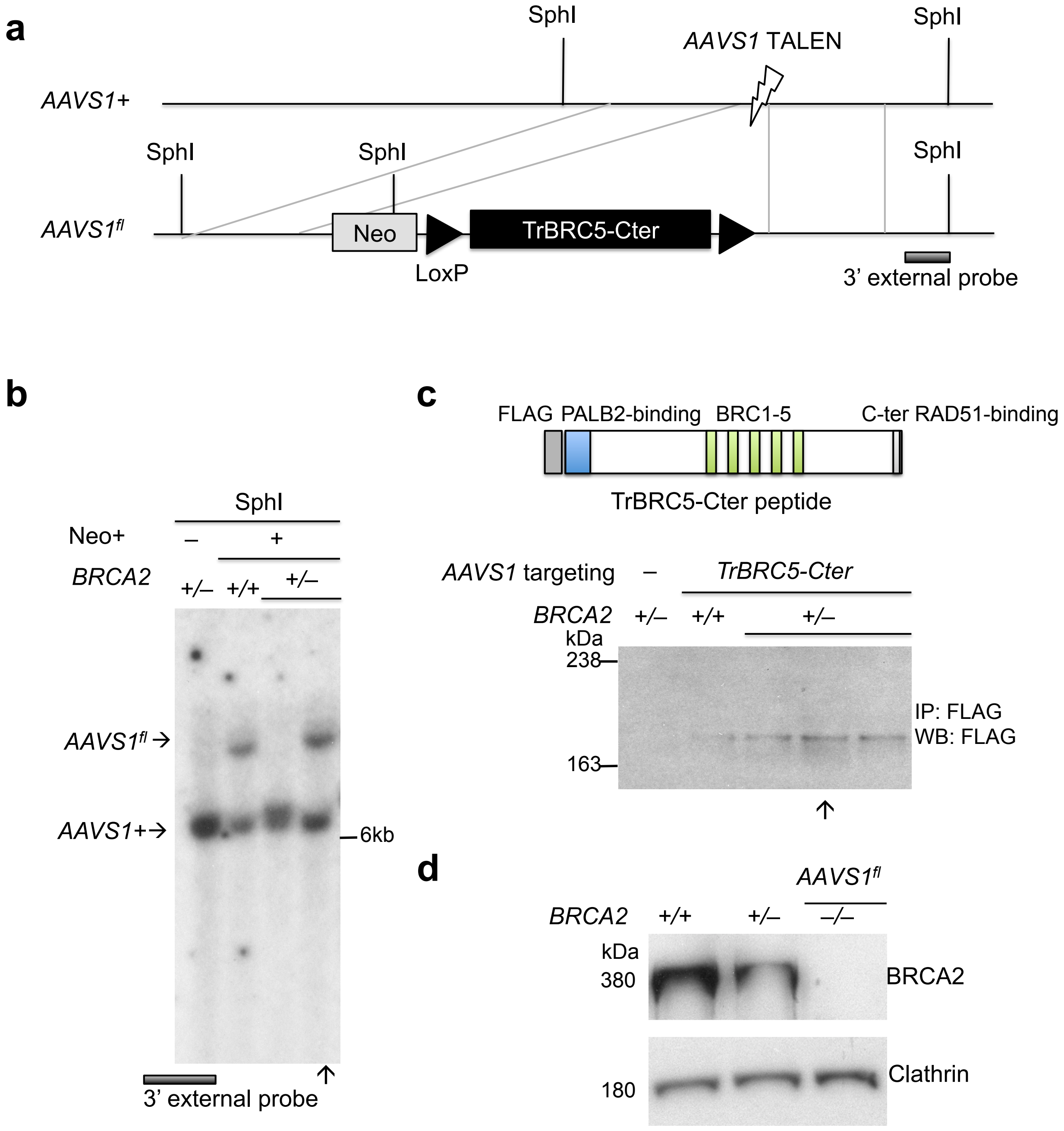

Supplementary Figure 2. Generation and characterization of *BRCA2*$^{-/-}$*AAVS1*^{fl} cells
The *BRCA2* null (-) alleles were generated using the Hygro donor fragment shown in **Supplementary Fig. 1a** and a cognate donor with a blasticidin-resistance gene replacing the hygromycin-resistance gene. The *AAVS1* targeting vector was introduced prior to generating the second *BRCA2* null allele.
a. Schematic of *AAVS1* targeting strategy. Restriction digestion sites and probes for Southern blotting are shown. Lightning symbol, *AAVS1* TALEN recognition sites; Neo, Neomycin-resistance gene cassette.
b. Southern blot to confirm correct gene targeting at the *AAVS1* locus in Neo+ clones. Vertical arrow indicates the clone chosen for subsequent experiments which has one targeted and one untargeted allele.
c. Western blot showing FLAG-tagged TrBRC5-Cter peptide expression by immuno-precipitation. Domain structure of the TrBRC-Cter peptide is shown at top. TrBRC5-Cter peptide expression is not detectible by

direct FLAG Western blotting (i.e., without enrichment by FLAG IP), suggesting low expression. Vertical arrow indicates the clone chosen for subsequent experiments.
d. Western blot showing loss of BRCA2 protein in *BRCA2–/–AAVS1*^{fl} cells.

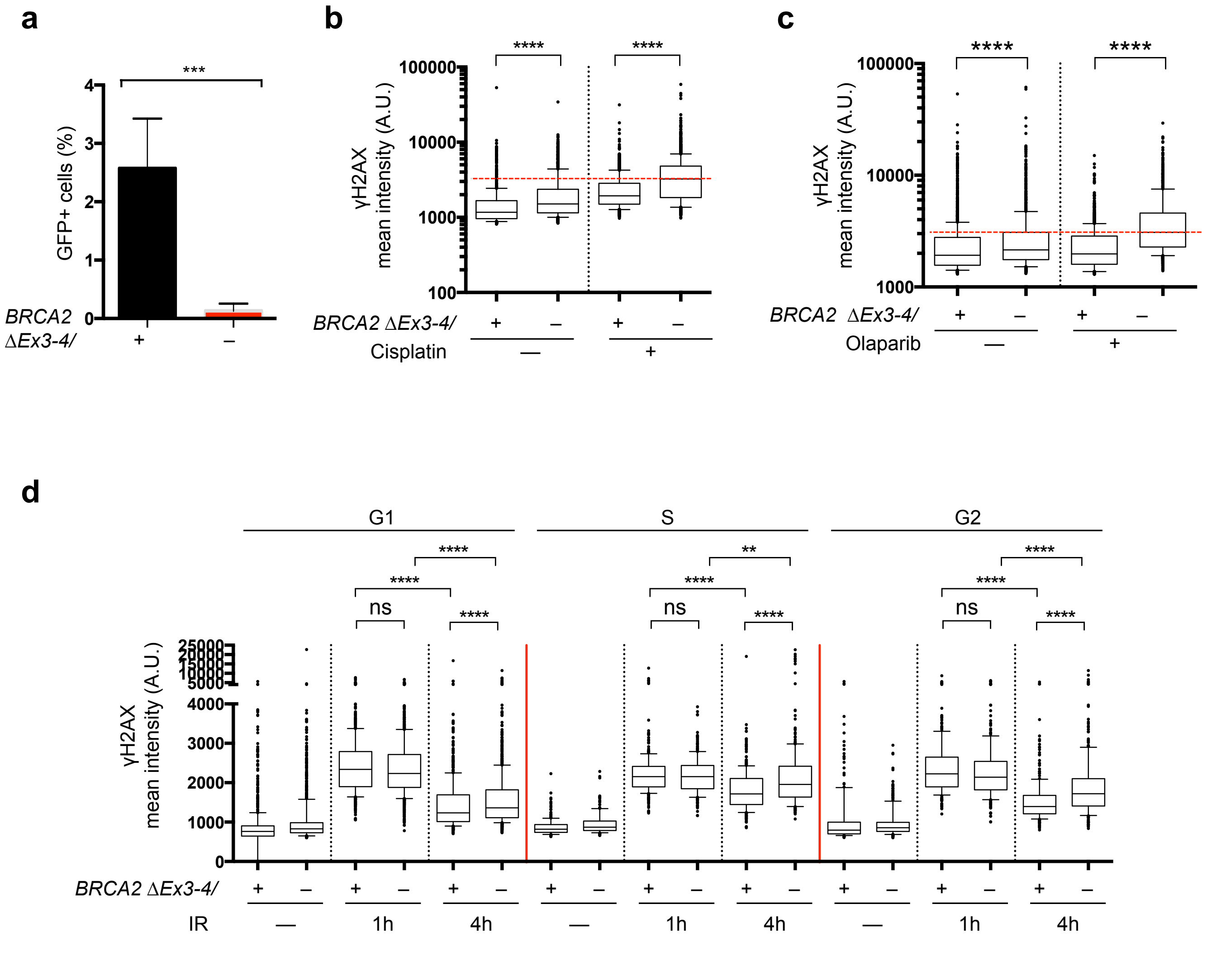

Supplementary Figure 3. BRCA2 inactivation causes HR deficiency

a. HR analysis. *BRCA2*$^{\Delta Ex3\text{-}4/-}$ cells were infected with I-SceI-expressing lentivirus and the percent GFP+ cells was analyzed by an unpaired two-tailed t test. n>3. Error bars, s.d.

b. Cisplatin-induced γH2AX. Cells were treated with 5 µM cisplatin for 5 h and released for another 24 h before analysis. γH2AX mean nuclear intensities of >1000 individual cells are shown from one experiment, which is representative of three independent experiments, analyzed by a two-tailed Mann-Whitney test. Box and whiskers show the 10th and 90th percentiles. The dotted red line indicates the median of the *BRCA2* mutant cells exposed to cisplatin.

c. Olaparib-induced γH2AX. Cells were treated with 5 µM olaparib for 24 h before analysis as in **b** (representative of two independent experiments). The dotted red line indicates the median of the *BRCA2* mutant cells exposed to olaparib.

d. IR-induced γH2AX. Cells were irradiated with 5 Gy and harvested 1 or 4 h later. EdU was added to all samples 1h before harvest for analysis. Cell cycle stages were determined based on EdU and DAPI intensity. γH2AX mean nuclear intensities of cells from each cell cycle stage are shown from one experiment, which is representative of two independent experiments, analyzed by a two-tailed Mann-Whitney test.

ns, not significant; **, p<0.01; ***, p<0.001; ****, p<0.0001.

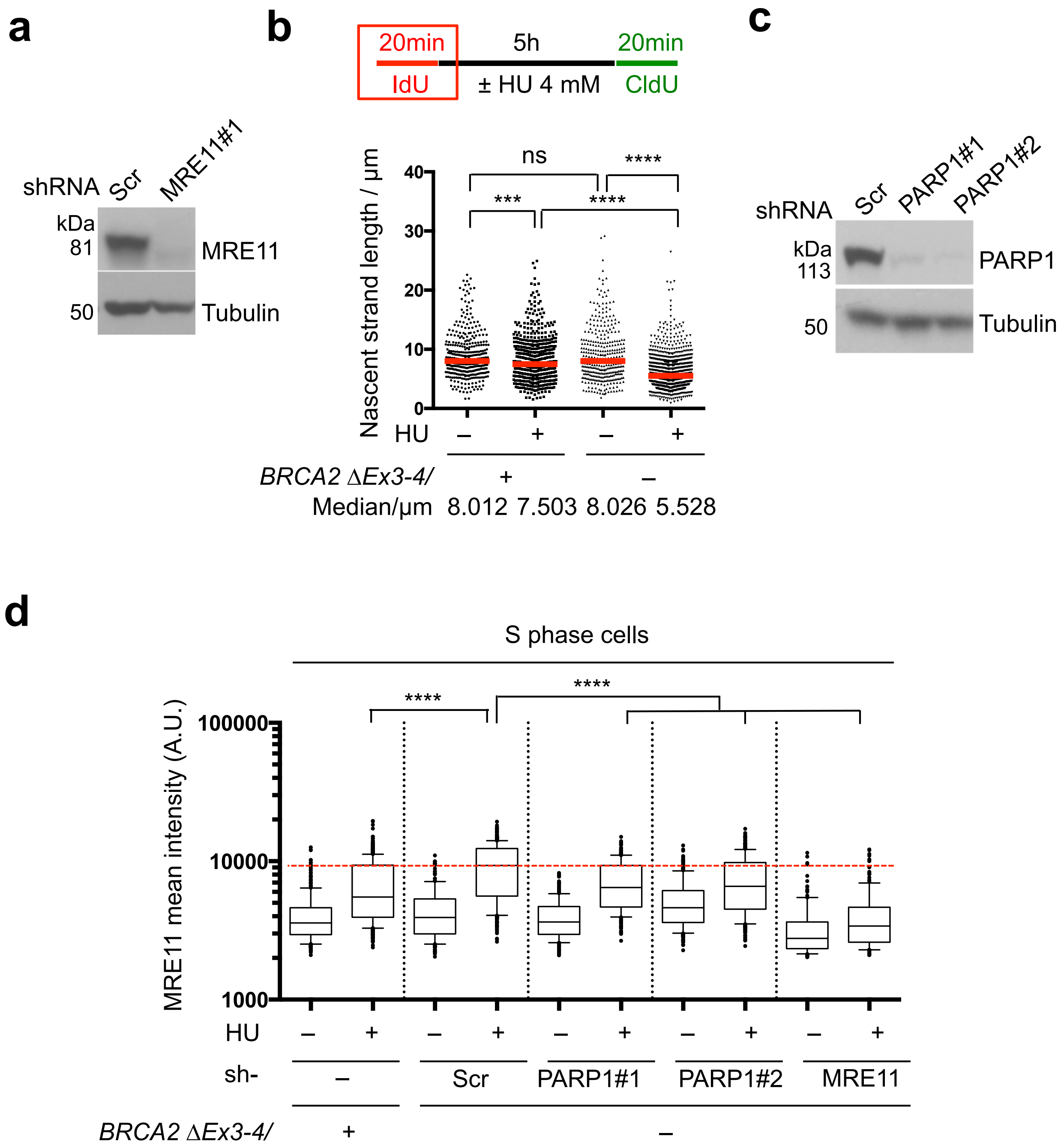

Supplementary Figure 4. PARP1 mediates MRE11 chromatin recruitment upon replication stress

a. Western blot showing MRE11 knockdown (related to Fig. 2b).

b. DNA fiber analysis to quantify fork protection with or without HU treatment. A schematic of the experimental design is shown (top). IdU tract length was quantified to indicate nascent strand length. The median IdU tract lengths are indicated (red bars). Graphs represent the pooled results of >200 fibers per genotype from two independent experiments.

c. Western blot showing PARP1 knockdown (related to Fig. 2b).

d. MRE11 chromatin recruitment assay. PARP1 knockdown cells were either treated with EdU together with HU (4 mM) for 5 h or EdU alone for 1h before pre-extraction and analyzed for chromatin-bound MRE11.

MRE11 mean nuclear intensities of EdU+ cells are shown from one experiment, which is representative of two independent experiments. The dotted red line indicates the median of the *BRCA2* mutant cells treated with the scrambled shRNA exposed to HU. Box and whiskers show the 10th and 90th percentiles.
ns, not significant; ***, $p<0.001$; ****, $p<0.0001$ (two-tailed Mann-Whitney test).

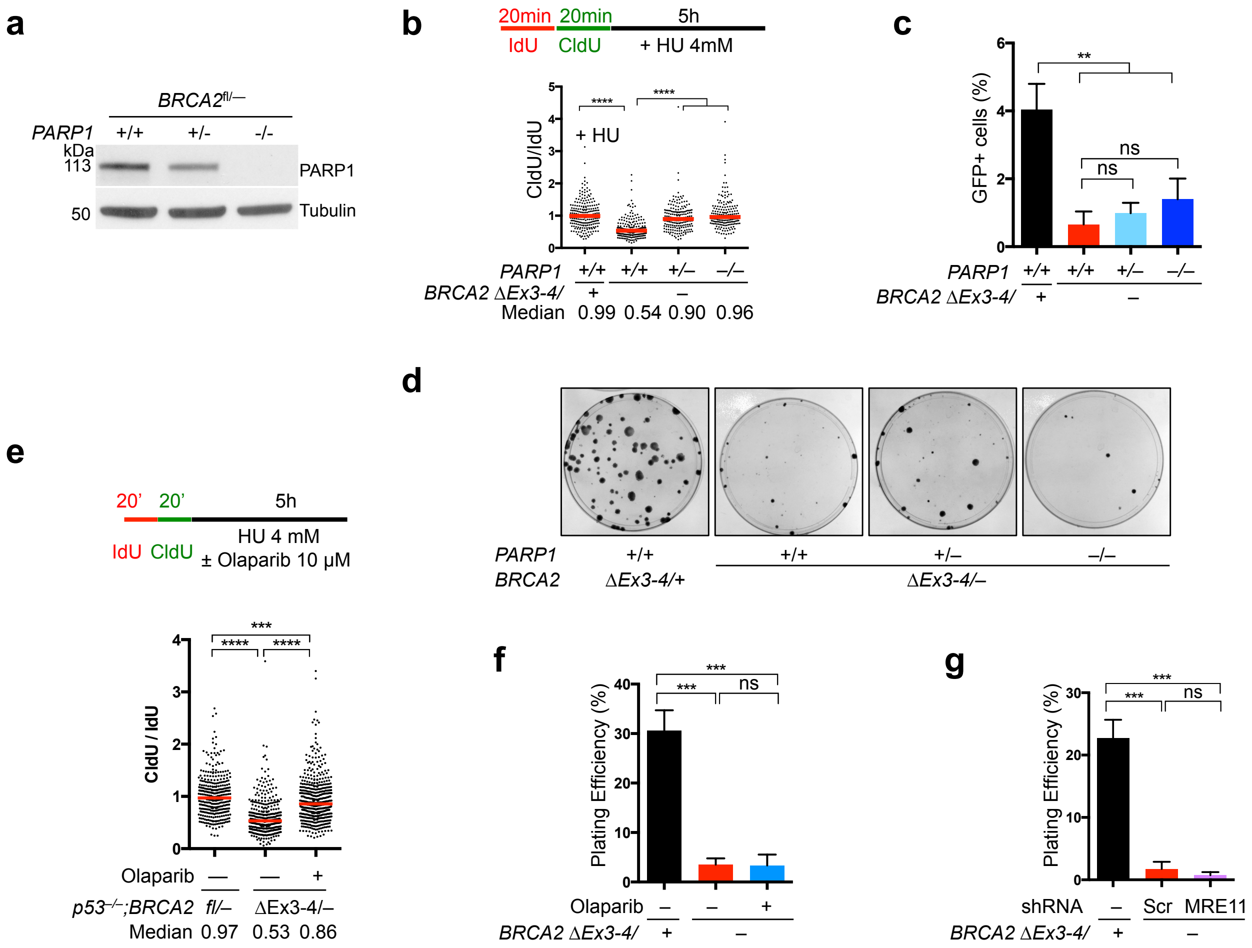

Supplementary Figure 5. Fork protection is not sufficient to support cell viability

a. Western blot showing PARP1 protein levels in indicated cells.

b. DNA fiber analysis to quantify fork protection in the presence of HU. Median CldU/IdU tract length ratios are indicated in the graph (red bars). Graphs represent the pooled results of >200 fibers per genotype from two independent experiments, analyzed by a two-tailed Mann-Whitney test.

c. HR analysis. Cells were infected with I-SceI-expressing lentivirus and the percent GFP+ cells was analyzed by an unpaired two-tailed t test. n=3.

d. Clonogenic survival. More than 40 of the residual colonies from each of the *BRCA2*$^{\Delta Ex3\text{-}4/–}$ plates were picked, and all were confirmed by PCR to have maintained the *BRCA2*$^{fl/–}$ genotype (i.e., escaped Cre recombination; primer design in **Supplementary Fig. 7a**).

e. DNA fiber analysis. *BRCA2*$^{fl/–}$ *p53*$^{–/–}$ and *BRCA2*$^{\Delta Ex3\text{-}4/–}$ *p53*$^{–/–}$ cells (derived from pre- and post-Cre colonies from **Fig. 3g**, respectively) were analyzed for fork protection in the presence of HU, and in the latter case olaparib, as shown in the schematic above. Median CldU/IdU tract length ratios are indicated in the graph (red bars). Graphs represent the pooled results of >300 fibers per sample from three independent experiments, analyzed by a two-tailed Mann-Whitney test.

f. Cells were treated without or with olaparib (10 µM, 3 h) prior to Cre expression and then were plated for clonogenic survival, analyzed by an unpaired two-tailed t test. n=3.

g. Clonogenic survival analysis using an unpaired two-tailed t test. n=3.

Error bars, s.d. ns, not significant; **, p<0.01; ***, p<0.001; ****, p<0.0001.

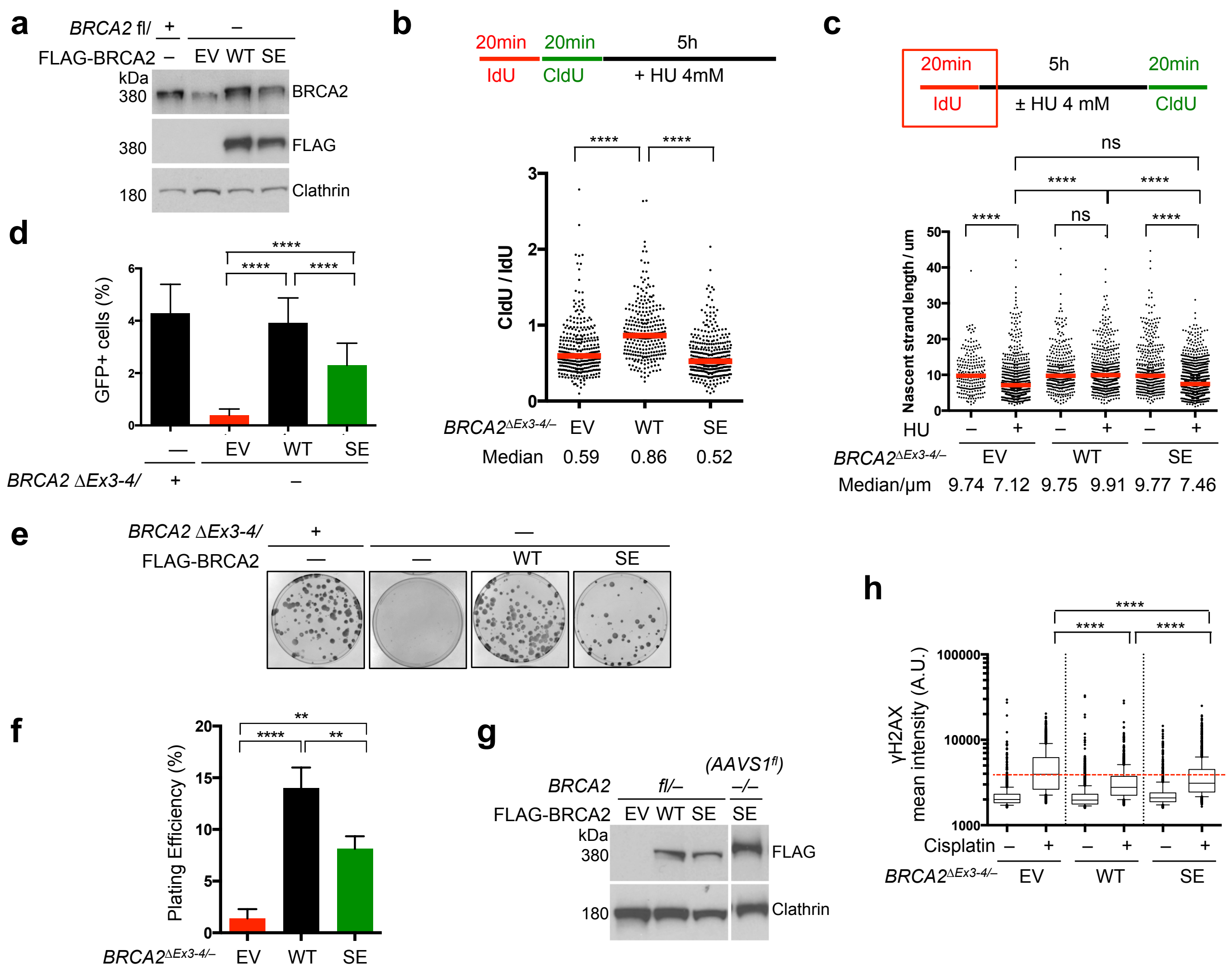

Supplementary Figure 6. Fork protection is not essential for cell viability or DNA repair

a. BRCA2 Western blot for *$BRCA2^{fl/–}$* cells stably expressing FLAG-tagged BRCA2 WT or BRCA2 SE, and *$BRCA2^{fl/+}$* cells as a control for endogenous BRCA2 protein level.

b,c. DNA fiber analysis of cells treated as shown at top to quantify fork protection. Median CldU/IdU tract length ratios (**b**) or IdU tract lengths (**c**) are indicated in the graph (red bars). Graphs represent the pooled results of >200 fibers per sample from at least two independent experiments, analyzed by a two-tailed Mann-Whitney test.

d. HR analysis. Cells were infected with I-SceI-expressing lentivirus and the percent GFP+ cells was analyzed by an unpaired two-tailed t test. n>3.

e, f. Clonogenic survival. Representative plates (**e**) and quantification of plating efficiency (**f**) are shown, analyzed by an unpaired two-tailed t test. n=4.

g. Western blot to compare stably transfected FLAG-tagged BRCA2 transgene expression in *$BRCA2^{fl/–}$* and *$BRCA2^{–/–}AAVS1^{fl}$* cells. Note the BRCA2 SE expression is higher in the *$AAVS1^{fl}$* system than in the *$BRCA2^{fl/–}$* system.

h. Cisplatin-induced γH2AX. Cells were treated with 5 μM cisplatin for 5 h and released for another 24 h before analysis. γH2AX mean nuclear intensities of >1000 individual cells are shown from one experiment, which is representative of two independent experiments, analyzed by a two-tailed Mann-Whitney test. Box and whiskers show the 10th and 90th percentiles. The dotted red line indicates the median of the *$BRCA2^{\Delta Ex3-4/–}$* cells exposed to cisplatin.

Error bars, s.d. ns, not significant; **, p<0.01; ****, p<0.0001.

a b

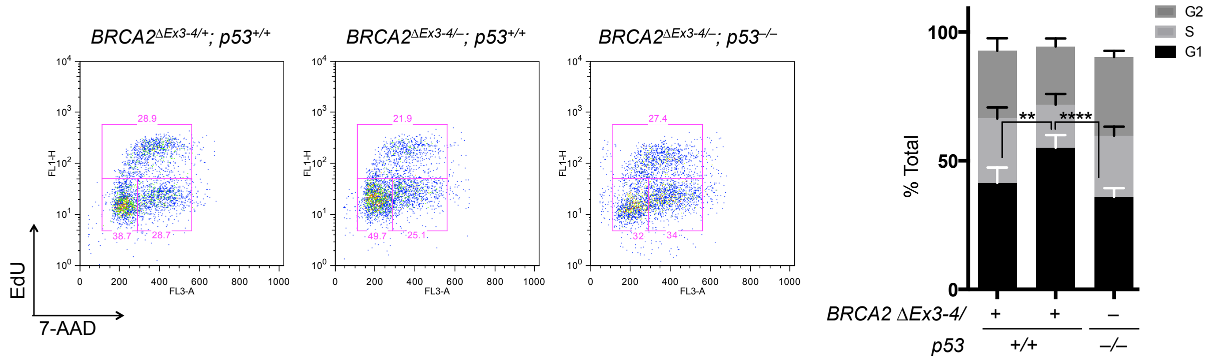

Supplementary Figure 7. BRCA2-deficient cells show p53-dependent G1 arrest

a, b. Cells were incubated with EdU for 30 min before cell cycle analysis based on EdU intensity and DNA content (7-AAD staining). Representative flow cytometry profiles (**a**) and quantification (**b**) are shown, analyzed by an unpaired two-tailed t test. n≥3. Error bars, s.d. **, $p<0.01$; ****, $p<0.0001$.

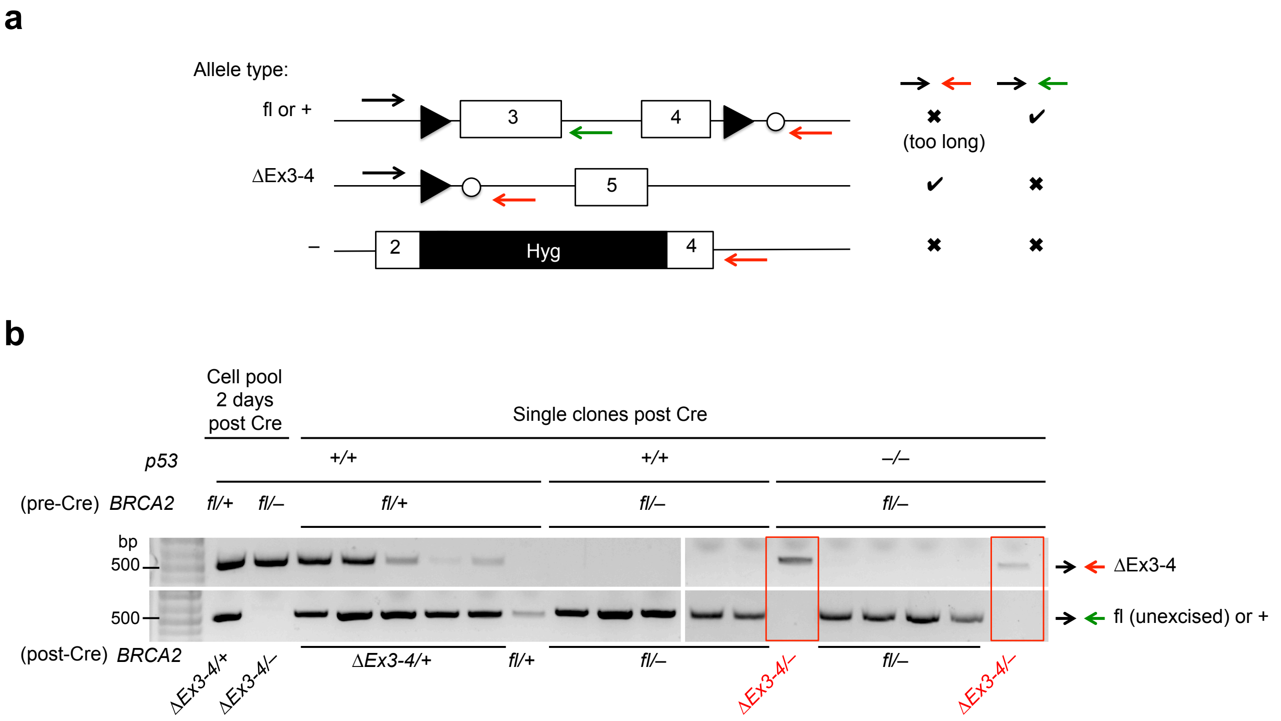

Supplementary Figure 8. Validated *BRCA2*$^{\Delta Ex3\text{-}4/–}$ clones form in the absence of p53

a. Schematic of primer design to distinguish the different *BRCA2* alleles.

b. Representative DNA gel image showing the results of genotyping PCR for the indicated cells. PCR was performed using either cell pools two days after Cre expression (first two lanes) or single colonies that grew after Cre expression. Primer sets used are shown on the right. The genotypes determined from PCR results are shown below the gel. The clones confirmed to be *BRCA2*$^{\Delta Ex3\text{-}4/–}$ are highlighted. Note that the only *BRCA2*$^{\Delta Ex3\text{-}4/–}$ clones are *p53*$^{–/–}$.

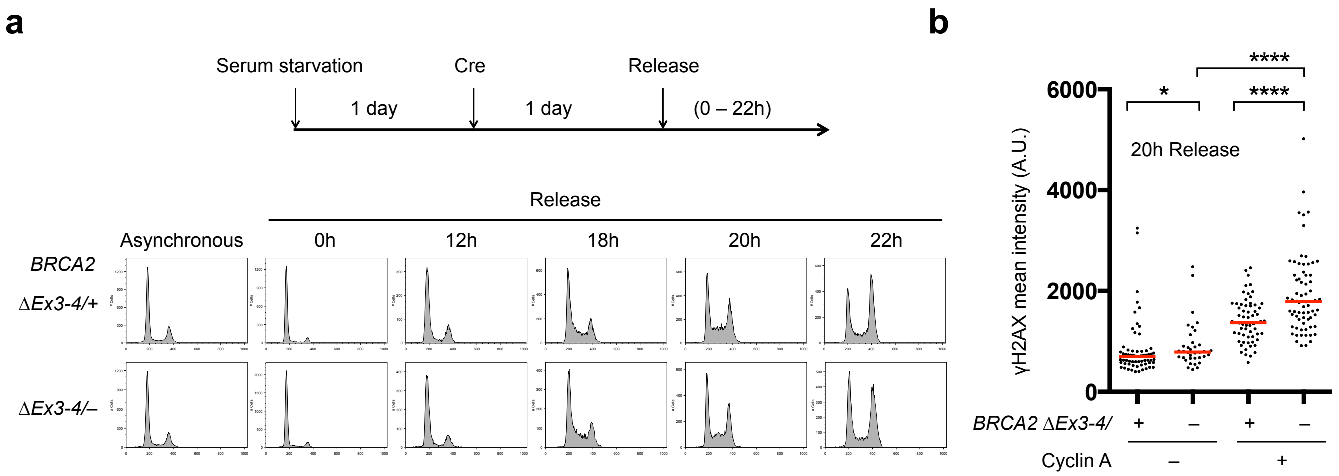

Supplementary Figure 9. S/G2-associated DNA damage induction upon BRCA2 deficiency

a. Cell cycle analysis of *BRCA2*$^{\Delta Ex3\text{-}4/–}$ cells released from serum starvation at time points indicated in the schematic at top.

b. Cells released for 20 h in **Fig. 4a** were also analyzed for γH2AX mean nuclear intensity in cyclin A– and cyclin A+ cells. Median γH2AX intensity, red bars. γH2AX mean nuclear intensities are shown from one experiment, which is representative of two independent experiments, analyzed by a two-tailed Mann-Whitney test. *, $p<0.05$; ****, $p<0.0001$.

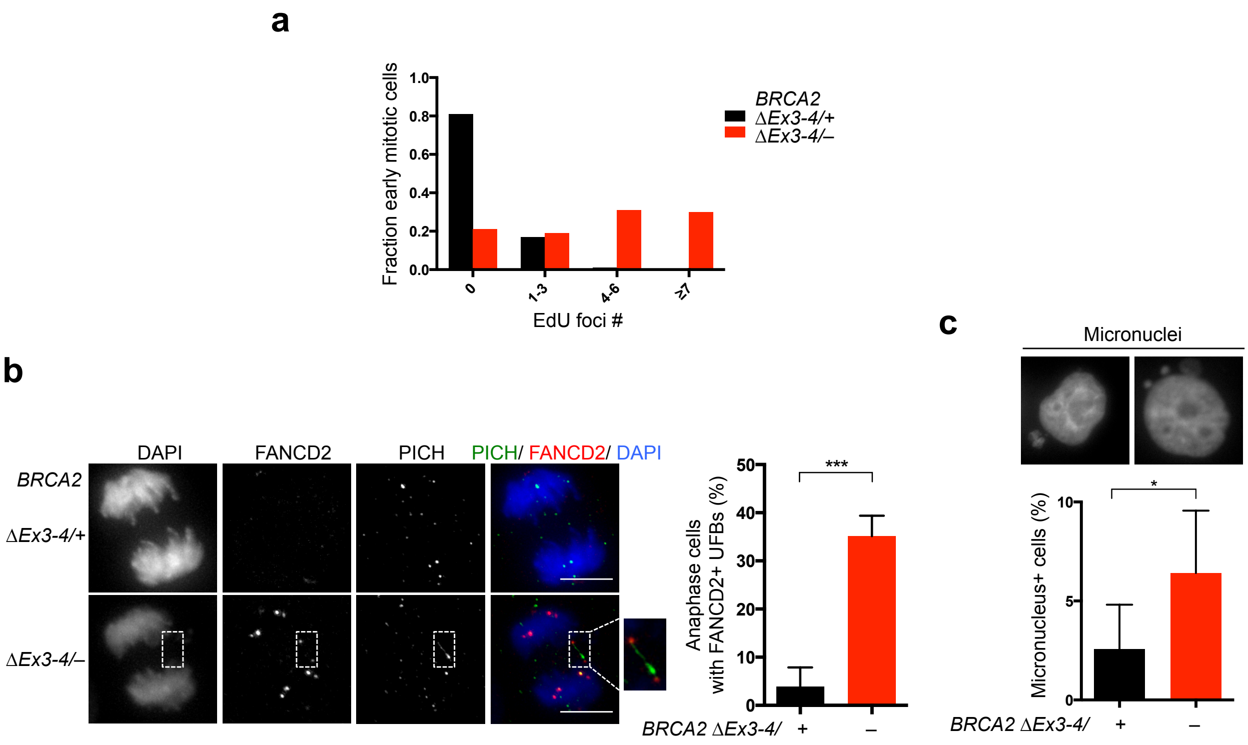

Supplementary Figure 10. BRCA2 deficiency causes under replication and mitotic abnormalities
a. EdU foci distribution quantification of samples from **Fig. 5a** (pooled results of three independent experiments).
b. Anaphase cells were analyzed for UFBs with PICH staining. Representative deconvolved images with an inset magnifying the UFB flanked by FANCD2 foci (left) and quantification (right) are shown. n=3. Scale bars, 10 µm.
c. Percent micronucleus-containing cells. Representative images are shown (top). n>3. Scale bars, 10 µm. Error bars, s.d. *, $p<0.05$; ***, $p<0.001$ (unpaired two-tailed t test).

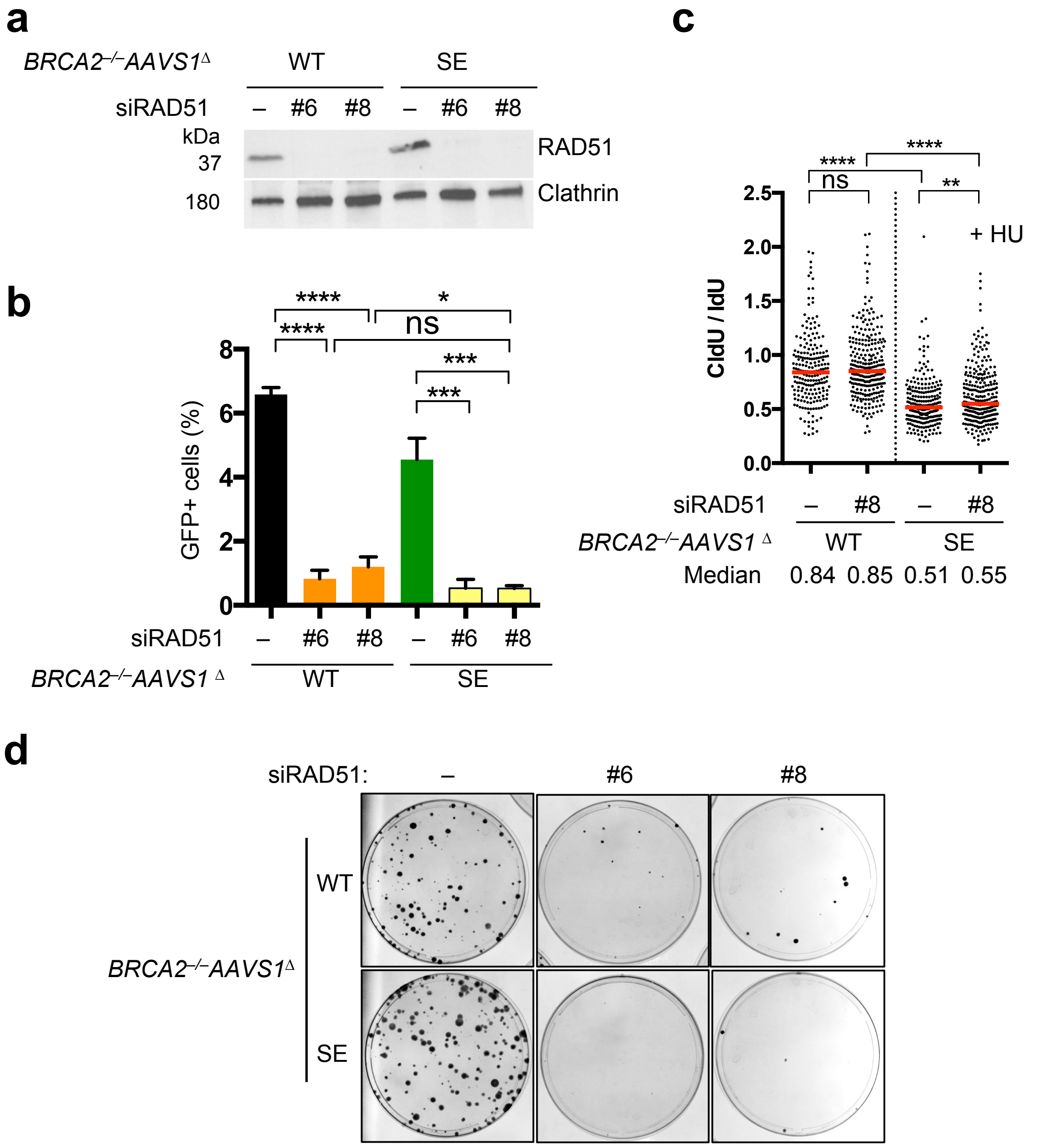

Supplementary Figure 11. HR is critical for cell viability

a. Western blot showing RAD51 knockdown in *BRCA2*$^{-/-}$*AAVS1*$^{\Delta}$ cells stably expressing BRCA2 WT or BRCA2 SE.

b. HR analysis. Cells expressing RAD51 siRNAs were infected with I-SceI-expressing lentivirus and the percent GFP+ cells was analyzed by an unpaired two-tailed t test. n=3. Error bars, s.d.

c. Cells expressing RAD51 siRNAs were analyzed for fork protection in the presence of HU. Median CldU/IdU tract length ratios are indicated in the graph (red bars). Graphs represent the pooled results of > 200 fibers per genotype from two independent experiments, analyzed by a two-tailed Mann-Whitney test.

d. RAD51 depletion in *BRCA2*$^{-/-}$*AAVS1*$^{\Delta}$ cells stably expressing BRCA2 WT or BRCA2 SE leads to a severe reduction in clonogenic survival.

ns, not significant; *, $p<0.05$; **, $p<0.01$; ***, $p<0.001$; ****, $p<0.0001$.

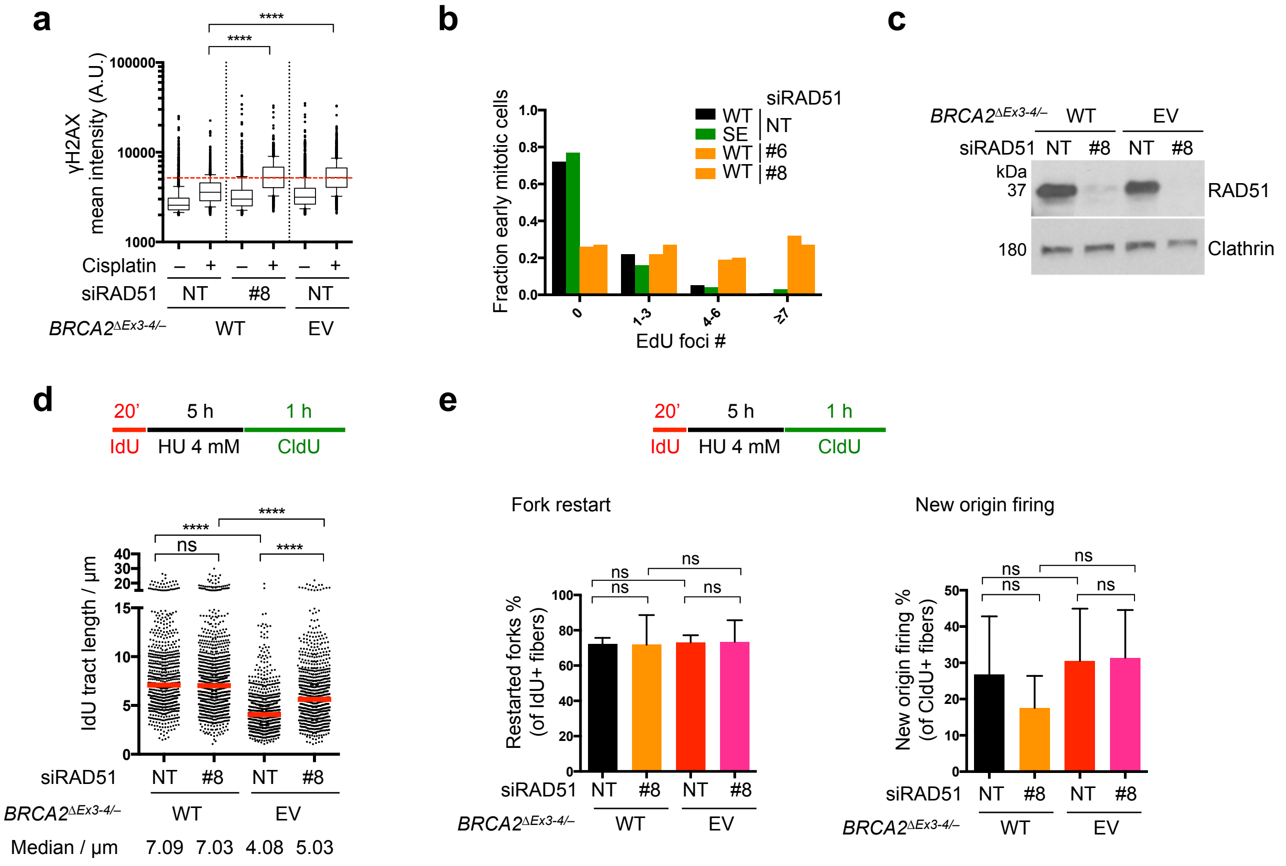

Supplementary Figure 12. Effects of RAD51 depletion on BRCA2-deficient cells

a. Cisplatin-induced γH2AX. Cells were treated with 5 μM cisplatin for 5 h and released for another 24 h before analysis. γH2AX mean nuclear intensities of >1000 individual cells are shown from one experiment, which is representative of three independent experiments, analyzed by a two-tailed Mann-Whitney test. Box and whiskers show the 10th and 90th percentiles. The dotted red line indicates the median of the NT (non targeting) siRNA-treated, EV-transfected *BRCA2*$^{\Delta Ex3\text{-}4/–}$ cells exposed to cisplatin.

b. EdU foci distribution of samples from **Fig. 7d** (pooled results of three independent experiments).

c. Western blots showing RAD51 knockdown in indicated cells.

d. DNA fiber analysis to quantify fork protection with HU treatment. A schematic of the experimental design is shown (top). Median IdU tract lengths are indicated in the graph (red bars). Graphs represent the pooled results of > 600 fibers per genotype from four independent experiments, analyzed by a two-tailed Mann-Whitney test.

e. DNA fiber analysis to quantify fork restart with HU treatment. A schematic of the experimental design is shown (top). Frequency quantifications of restarted forks (lower left) and newly fired origins (lower right) are shown, analyzed by an unpaired two-tailed t test. n=4. Error bars, s.d.

ns, not significant; ****, p<0.0001.

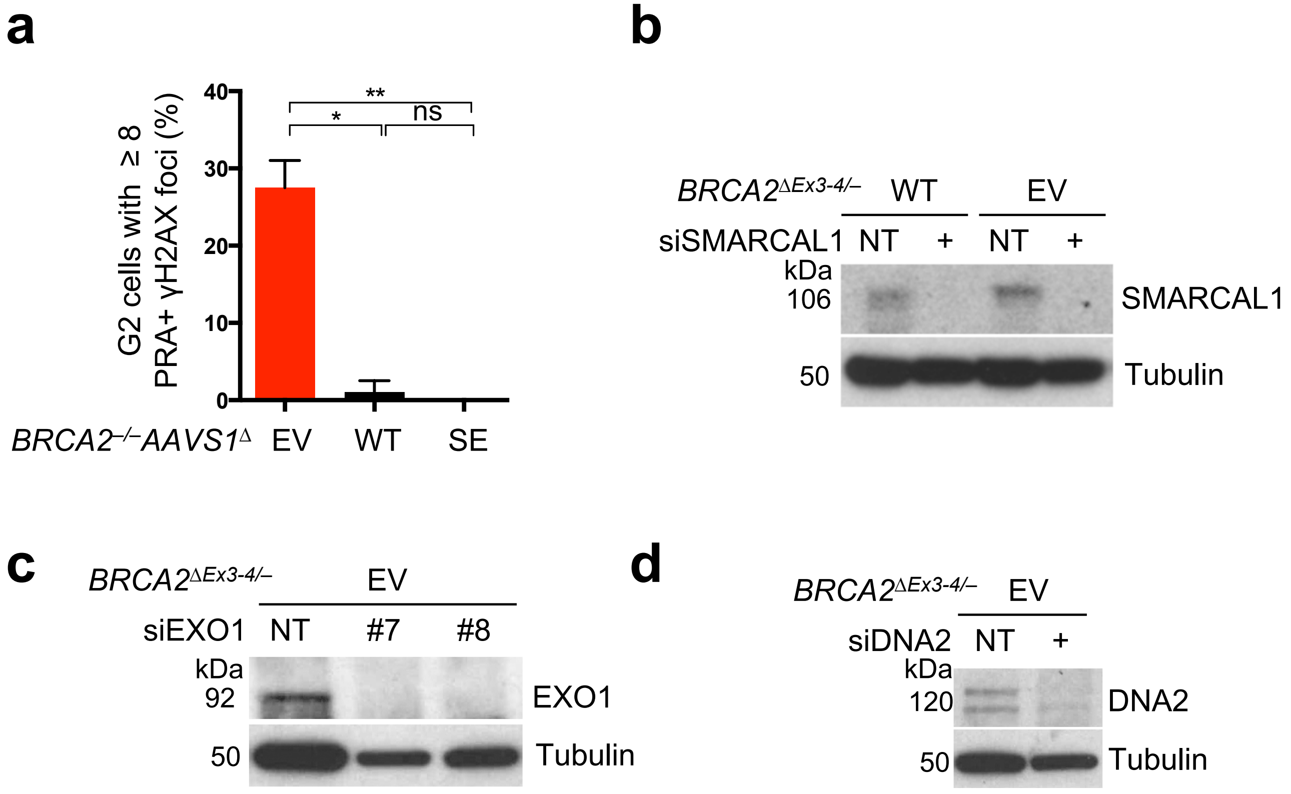

Supplementary Figure 13. Quantification of G2 lesions

a. *BRCA2*$^{-/-}$*AAVS1*$^{\Delta}$ cells complemented with BRCA2 expression vectors were incubated with EdU for 30 min before quantification of RPA+ γH2AX foci in G2 cells (EdU–, 2N DNA content) cells, analyzed by an unpaired two-tailed t test. n=2. Error bars, s.d.

b-d. Western blot showing SMARCAL1 (**b**), EXO1 (**c**) and DNA2 (**d**) knockdown.

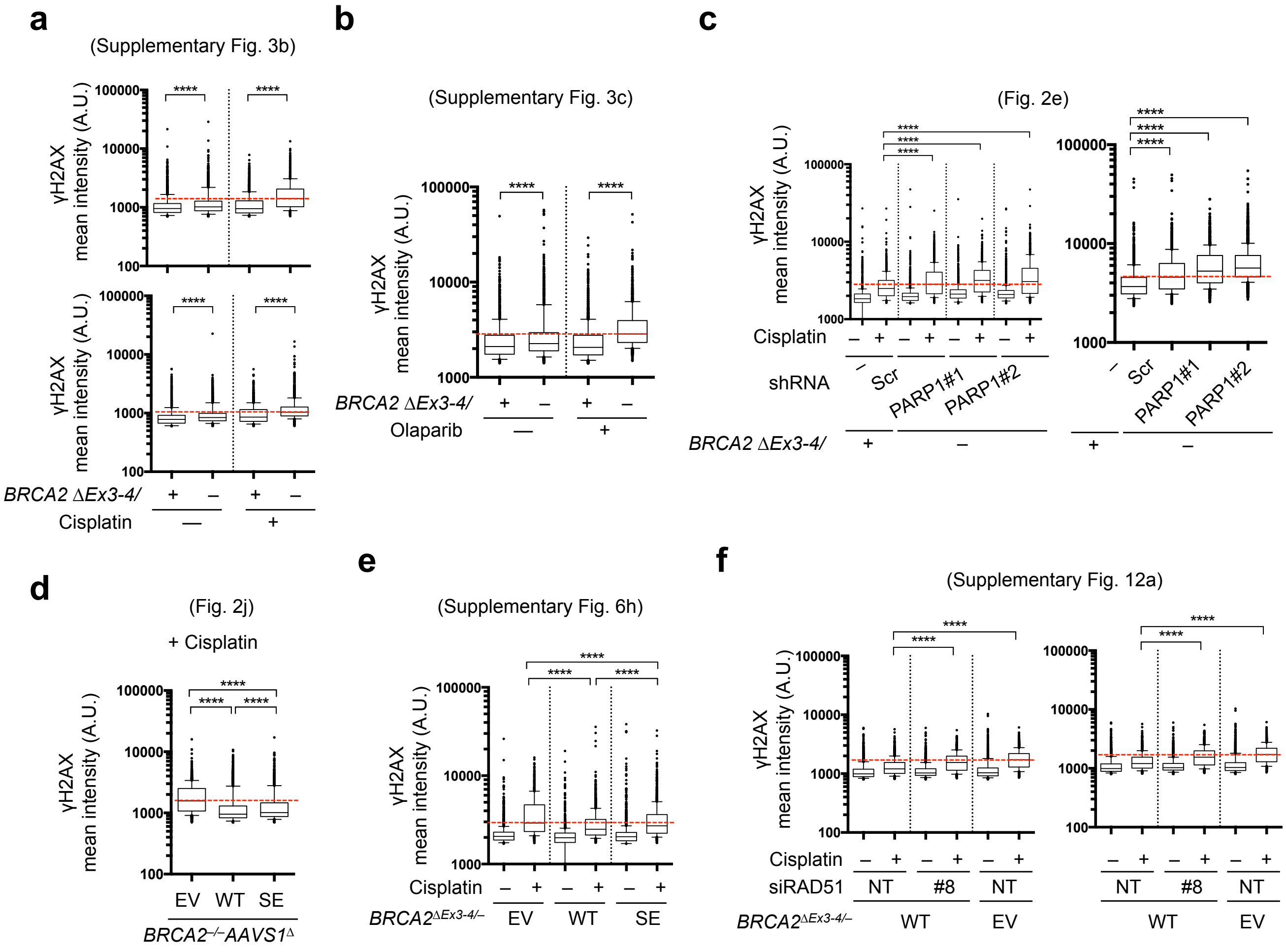

Supplementary Figure 14. γH2AX intensity replicates for the indicated figures

Fig. 1b: BRCA2 (top) and Clathrin (bottom)

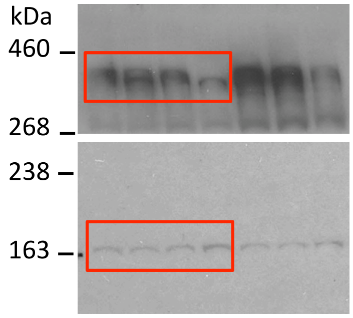

Fig. 1e: BRCA2 (top), FLAG (middle) and Clathrin (bottom)

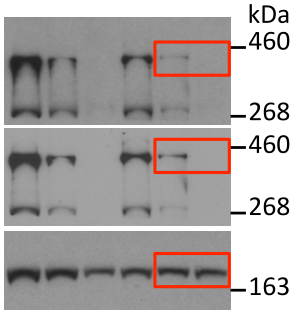

Fig. 2f: BRCA2 (top), FLAG (middle) and Clathrin (bottom)

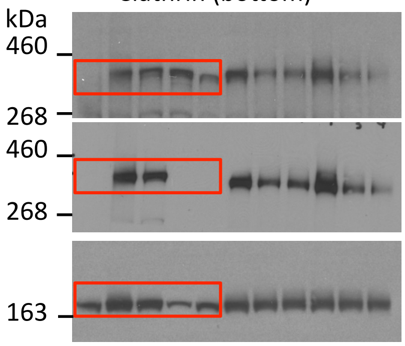

Fig. 3c: p53(top) and tubulin (bottom)

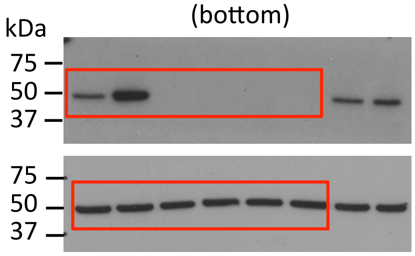

Fig. 3d: p53 (top), p21 (middle) and HDAC2 (bottom)

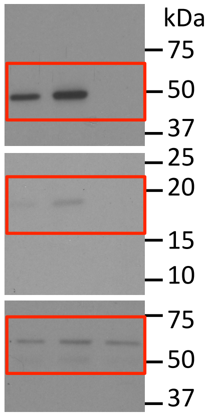

Fig. 7a: RAD51 (top) and Clathrin (bottom)

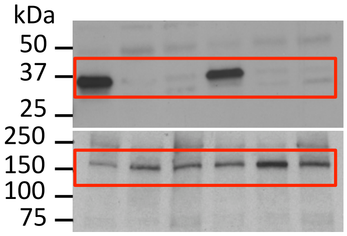

Supplementary Fig. 1b: Southern blot

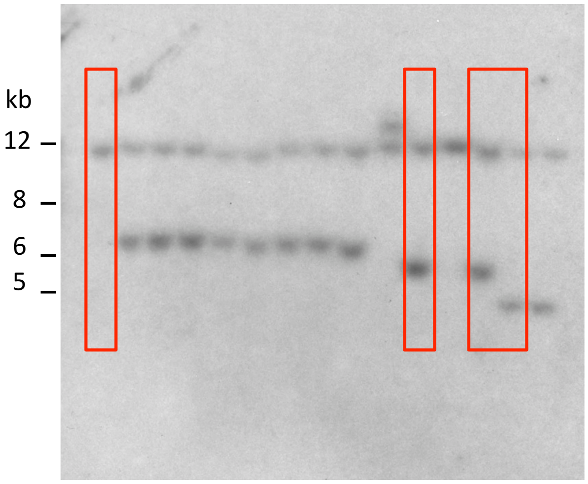

Supplementary Fig. 1c: Southern blot

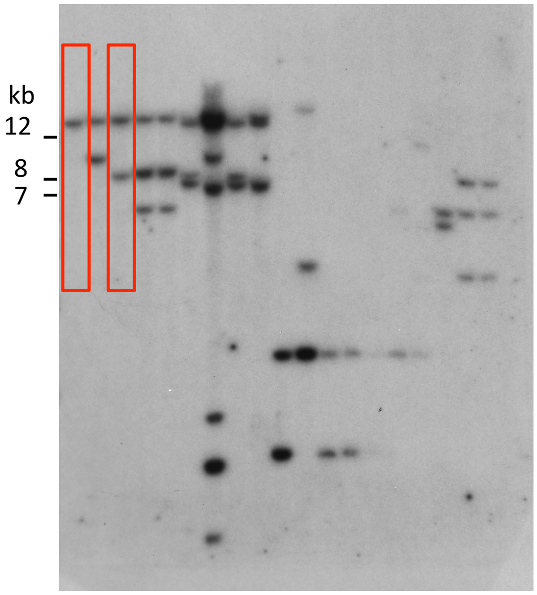

Supplementary Fig. 1d: Southern blot

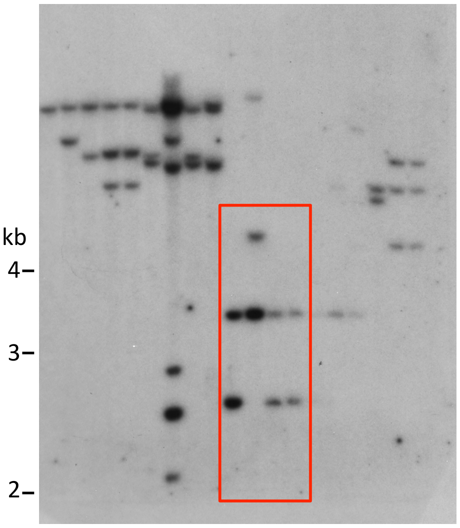

Supplementary Fig. 1g: BRCA2 *ΔEx3-4/+* (left) and *ΔEx3-4/–* (right) cells

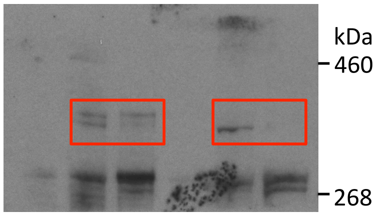

Supplementary Fig. 1g: HDAC2 *ΔEx3-4/+* (left) and *ΔEx3-4/–* (right) cells

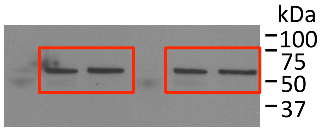

Supplementary Fig. 1g: Histone H3 *ΔEx3-4/+* (left) and *ΔEx3-4/–* (right) cells

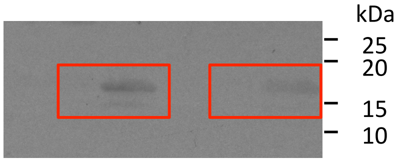

Supplementary Fig. 2b: Southern blot

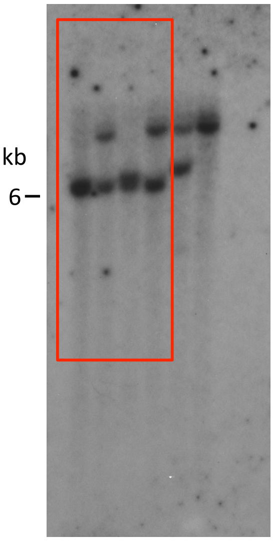

Supplementary Fig. 2c: FLAG IP WB

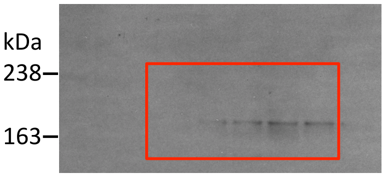

Supplementary Fig. 2d: BRCA2 (top) and Clathrin (bottom)

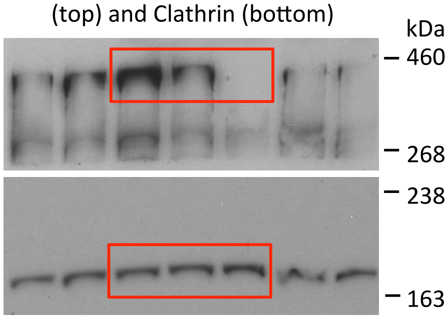

Supplementary Fig. 4a: MRE11 (top) and tubulin (bottom)

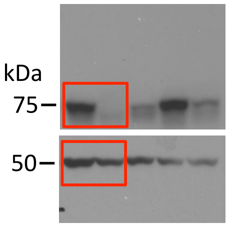

Supplementary Fig. 4c: PARP1 (top) and tubulin (bottom)

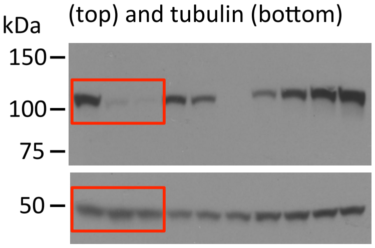

Supplementary Fig. 5a: PARP1 (top) and tubulin (bottom)

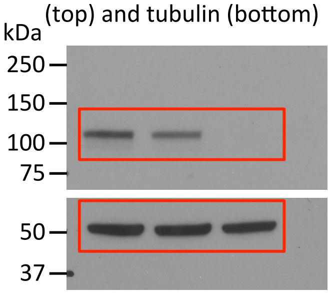

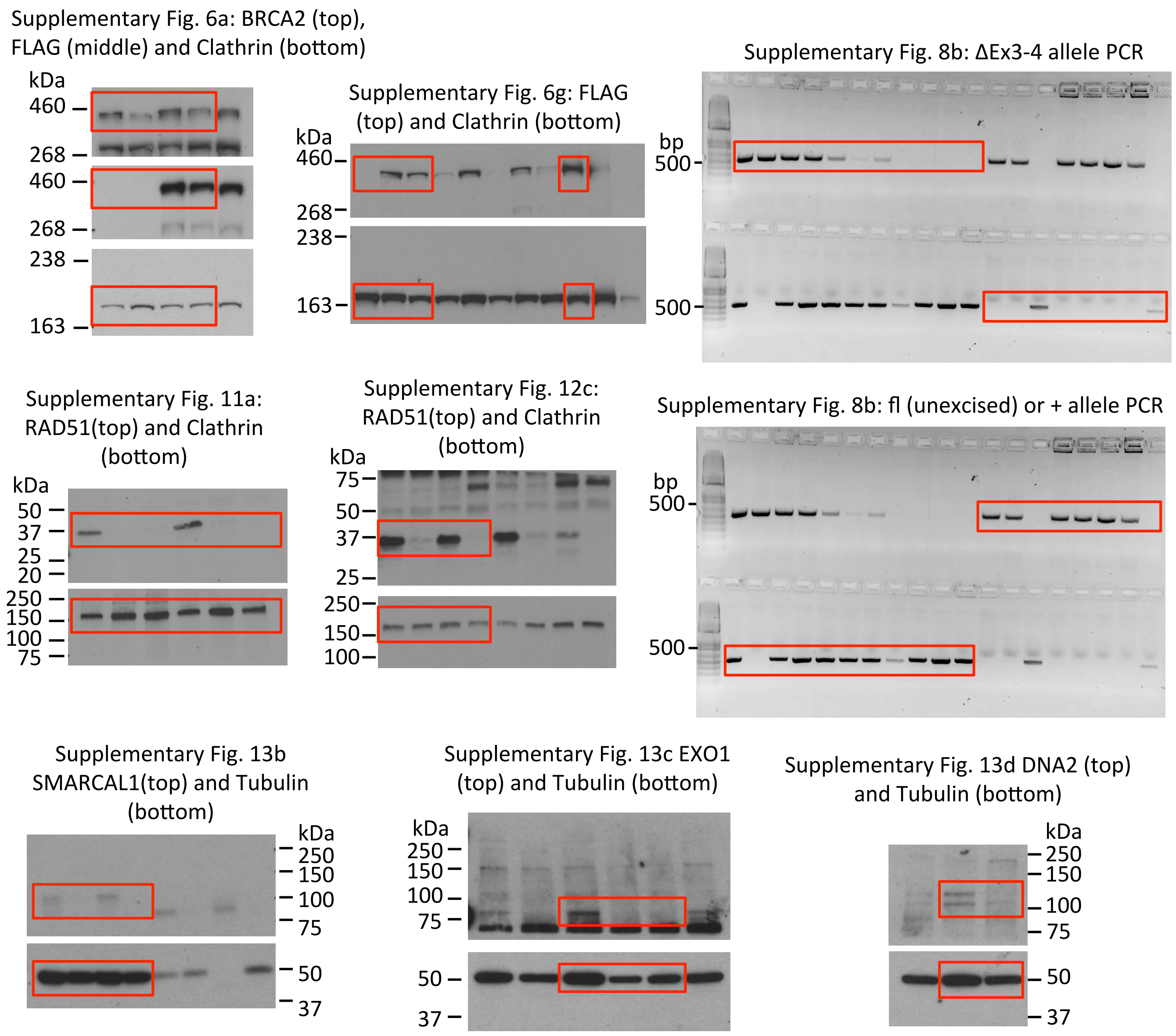

Supplementary Figure 15. Uncropped blots and gels
